# Inferring neural signalling directionality from undirected structural connectomes

**DOI:** 10.1038/s41467-019-12201-w

**Published:** 2019-09-19

**Authors:** Caio Seguin, Adeel Razi, Andrew Zalesky

**Affiliations:** 1 Melbourne Neuropsychiatry Centre, The University of Melbourne and Melbourne Health, Melbourne, VIC 3010 Australia; 2 Turner Institute for Brain and Mental Health, Monash University, Clayton, VIC 3800 Australia; 3 The Wellcome Trust Centre for Neuroimaging, University College London, London, WC1E 6BT UK; 4 Department of Electronic Engineering, NED University of Engineering and Technology, Karachi, Sindh 75270 Pakistan; 5 Department of Biomedical Engineering, Melbourne School of Engineering, The University of Melbourne, Melbourne, VIC 3010 Australia

**Keywords:** Network models, Neural circuits, Applied mathematics, Computer science

## Abstract

Neural information flow is inherently directional. To date, investigation of directional communication in the human structural connectome has been precluded by the inability of non-invasive neuroimaging methods to resolve axonal directionality. Here, we demonstrate that decentralized measures of network communication, applied to the undirected topology and geometry of brain networks, can infer putative directions of large-scale neural signalling. We propose the concept of send-receive communication asymmetry to characterize cortical regions as senders, receivers or neutral, based on differences between their incoming and outgoing communication efficiencies. Our results reveal a send-receive cortical hierarchy that recapitulates established organizational gradients differentiating sensory-motor and multimodal areas. We find that send-receive asymmetries are significantly associated with the directionality of effective connectivity derived from spectral dynamic causal modeling. Finally, using fruit fly, mouse and macaque connectomes, we provide further evidence suggesting that directionality of neural signalling is significantly encoded in the undirected architecture of nervous systems.

## Abstract (web summary)

Neural signalling is directional, but non-invasive neuroimaging methods are unable to map directed connections between brain regions. Here, the authors show how network communication measures can be used to infer signalling directionality from the undirected topology of brain structural connectomes.

## Introduction

Understanding how the structural substrate of connectomes^1,2^ gives rise to the rich functional dynamics observed in nervous systems is a major goal in neuroscience^3–6^. Anatomical connectivity constrains and facilitates neural information transfer, which in turn gives rise to synchronization (i.e., functional connectivity) between neural elements. Therefore, knowledge of how neural signals are communicated in nervous systems can establish a bridge between structural and functional descriptions of brain networks^7–10^.

While information can be directly communicated between anatomically connected neural elements, polysynaptic communication is needed for structurally unconnected elements. Network communication models describe a propagation strategy that delineates the signaling pathways utilized to transfer information between network nodes. In turn, a network communication measure quantifies the *communication efficiency* along the identified pathways from a graph-theoretic standpoint. Efficient communication pathways are generally short, traverse few synapses and comprise strong and reliable connections^11^.

Several network models of polysynaptic communication have been proposed^9^. Shortest paths routing is the most ubiquitous model^12–14^, which proposes that communication occurs via optimally efficient routes. However, the identification of shortest paths mandates global knowledge of network topology^8,9,15,16^. This requirement is unlikely to be met in biological systems, in which individual elements (e.g., neurons or brain regions) do not possess information on all connections comprising the network. This has motivated research on decentralized models that capitalize on local knowledge of network properties to facilitate signaling. Examples include navigation^15,17,18^, spreading dynamics^19,20^, and diffusion processes^21–24^.

**Fig. 1:**
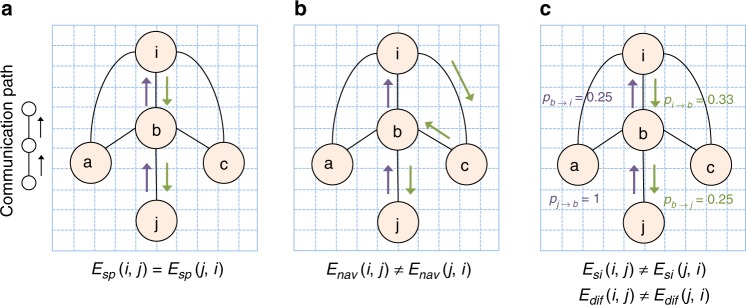
Illustrative examples of send-receive communication asymmetry. The toy network is spatially embedded, unweighted and undirected. Communication efficiency from node *i* to *j* under measure *x* ∈ {*sp*, *nav*, *si*, *dif*} is denoted *E*_*x*_(*i*, *j*). Shortest path and navigation efficiencies are computed as the inverse of the number of connections comprising shortest and navigation paths, respectively. Diffusion efficiency relates to how quickly, on average, a random walker can travel between two nodes, while search information relates to the probability that a random walker will travel between two nodes via the shortest path linking them. The path identified under each communication model is designated with green (*i* → *j*) and mauve (*j* → *i*) arrows. Send-receive communication asymmetry refers to *E*_*x*_(*i*, *j*) ≠ *E*_*x*_(*j*, *i*). **a** Shortest path efficiency is always symmetric in undirected networks, and thus *E*_*sp*_(*i*, *j*) = *E*_*sp*_(*j*, *i*). **b** Navigation routes information by progressing to the next directly connected node that is closest in distance to the target node. This results in the *i-c-b-j* and *j-b-i* navigation paths, with respective efficiency *E*_*nav*_(*i*, *j*) = 0.33 and *E*_*nav*_(*j*, *i*) = 0.5. Hence, navigation is more efficient from node *j* to node *i*. **c** Arrows denote the symmetric shortest paths between *i* and *j*. Arrows are annotated with the probabilities that a random walker will traverse their respective connections based on node degree (e.g., each of the 3 connections of node *i* has approximately 0.33 probability to be traversed by a random walker leaving *i*). We have *E*_*si*_(*i*, *j*) ∝ 0.33 × 0.25 = 0.0825 and *E*_*si*_(*j*,*i*) ∝ 1 × 0.25 = 0.25. Hence, a random walker has higher probability of traveling via the shortest path in the *j* → *i* direction, characterizing search information asymmetry between *i* and *j*. Similarly, on average, a random walker is expected to visit fewer nodes traveling from *i* to *j* than from *j* to *i*. Hence, *E*_*dif*_(*j*, *i*) > *E*_*dif*_(*i*, *j*), characterizing diffusion efficiency asymmetry

Many decentralized communication models are asymmetric^8,15,16,19^, meaning that sending information from region *i* to region *j* can be performed more efficiently than sending information from region *j* to region *i*. We coin the term *send-receive communication asymmetry*, or simply *send-receive asymmetry* to describe this property. Importantly, the interplay between decentralized communication, network topology and possibly geometry can result in communication asymmetry in undirected networks (Fig. 1). This provides an opportunity to infer putative directions of information flow from current descriptions of the human structural connectome, for which knowledge about the directionality of individual connections is unknown due to inherent limitations of in vivo diffusion imaging. Therefore, decentralized network communication measures may help bridge the gap between our symmetric understanding of human connectome structure and the ample evidence for its asymmetric functional dynamics^25–27^.

We provide multiple lines of evidence suggesting that decentralized communication measures, applied to undirected brain networks, can provide new insight into the directionality of neural information flow. We begin by classifying individual cortical regions and subsystems as senders (biased towards the efficiency of outgoing paths), neutral (symmetric communication efficiency) and receivers (biased towards the efficiency of incoming paths). We demonstrate that regional variation in send-receive asymmetry recapitulates established hierarchies of cortical organization. Next, we analyze pairwise send-receive asymmetries between cortical subsystems, providing multi-scale maps of communication directionality in the human cortex. Crucially, we validate these maps by showing a significant association between send-receive asymmetry and directionality of effective connectivity derived from dynamic causal modeling (DCM) applied to resting-state functional magnetic resonance imaging (fMRI). These results suggest that the undirected topology of the human connectome imposes constraints on neural signaling directionality. We further investigate this notion by examining fly, mouse and macaque connectomes. We leverage the presence of directed connections in these brain networks to provide additional evidence that neural signaling directionality is not exclusively contingent on directed axonal projections, being partially shaped by the undirected organization of nervous systems.

## Results

### Measures of send-receive communication asymmetry

We investigated three asymmetric network communication measures: (i) navigation efficiency, (ii) diffusion efficiency, and (iii) search information (Methods section, and Network communication measures). Briefly, navigation efficiency^15^ relates to the length of paths identified by navigation or greedy routing^17,28^, with higher values of efficiency indicating faster and more reliable communication between nodes. Diffusion efficiency^23^ quantifies how many intermediate regions (synapses), on average, a naive random walker needs to traverse to reach a desired destination region. Finally, search information is related to the probability that a random walker will travel from one region to another via the shortest path between them^8,29^, quantifying the extent to which efficient routes are hidden in the network topology. Together, these measures are representative of different conceptualizations of decentralized network communication^9,16^, from single-path routing via geometric navigation to diffusive signaling unfolding along multiple network fronts.

Communication asymmetry is introduced by the decentralized character of certain network communication models (Fig. 1). Consider the flow of information from one region, termed the *source node*, to another region, termed the *target node*. If this source-target pair is not directly connected, information must flow via a polysynaptic path that traverses one or more intermediate nodes. Decisions on how signals are propagated through the connectome depend on the local topology around each node. Since source and target nodes occupy potentially different vicinities, communication may happen through distinct paths, and thus with different efficiency, depending on the direction of information flow. In contrast, centralized communication models such as shortest path routing always yield symmetric paths in undirected networks.

**Fig. 2:**
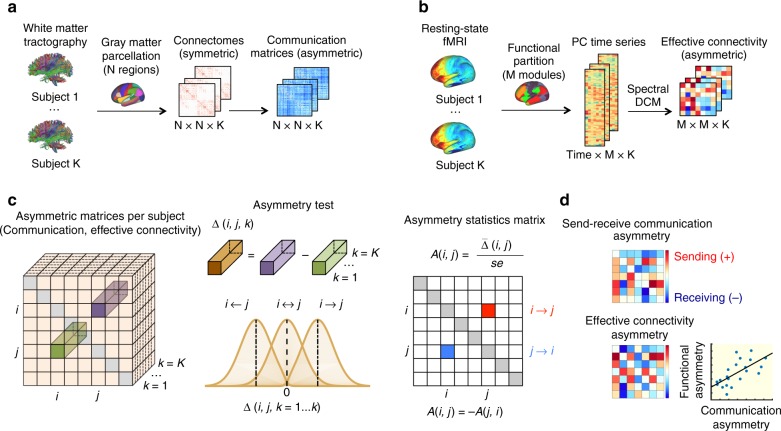
Methodology overview. **a** White matter tractography applied to diffusion MRI data for *K* = 200 adults participating in the HCP was used to map undirected (i.e., symmetric) weighted adjacency matrices representing the structural connectivity between *N* = 256,350,512 cortical regions. Navigation efficiency, diffusion efficiency and search information were computed between every pair of regions to generate asymmetric communication matrices. **b** Resting-state fMRI data for the same HCP participants was used to compute principal component (PC) time series summarizing the functional activity of *M* = 7,17,22 cortical subsystems. For each individual, effective connectivity between cortical subsystems was computed using spectral DCM. **c** Schematic of the communication asymmetry test. First, for a pair of nodes *i* and *j*, the difference in communication efficiency between the *i* → *j* and *j* ← *i* directions was computed. Performing this for *K* individuals yielded the distribution Δ(*i*, *j*, *k* = 1…*K*). Communication asymmetry was assessed by performing a one-sample *t*-test to determine whether the mean of this distribution is significantly different to 0, with *A*(*i*, *j*) defined as the resulting matrix of *t*-statistics. **d** The asymmetry test was applied to compute *M* × *M* matrices of communication and effective connectivity send-receive asymmetries between modules. We sought to test for correlations across the corresponding elements of these two matrices

We use *C* ∈ ℝ ^*N* × *N* × *K*^ to denote a set of communication matrices for *K* individuals, where *C*(*i*, *j*, *k*) denotes the communication efficiency from node *i* to node *j* for individual *k*, under an arbitrary communication measure (Fig. 2a). The difference in communication efficiency for opposing directions of information flow between *i* and *j* is given by Δ(*i*, *j*, *k*) = *C*(*i*, *j*, *k*) − *C*(*j*, *i*, *k*). We perform a one-sample *t*-test to determine whether the mean of the distribution Δ(*i*, *j*, *k* = 1…*K*) is significantly different to 0 (Fig. 2c). This yields a t-statistic, termed *A*(*i*, *j*), which quantifies the extent of communication asymmetry between *i* and *j*. In particular, if *A*(*i*, *j*) is significant and greater than zero, we conclude that communication can occur more efficiently from node *i* to node *j*, rather than from node *j* to node *i*. Note that *A*(*i*, *j*) = −*A*(*j*, *i*), and thus if *A*(*j*, *i*) is significantly less than zero, we reach the same conclusion. Repeating this test independently for all pairs of nodes yields the communication asymmetry matrix *A*, for which values are symmetric about the main diagonal, but with opposite signs.

The above measure is specific to pairs of nodes. We use a variation of this test to compute regional send-receive communication asymmetry by taking into account all outgoing and incoming communication paths of a given node (Methods section, Communication asymmetry test, and Supplementary Fig. 1). Regions that show a significantly higher efficiency of outgoing (incoming) communication are classified as putative senders (receivers), while nodes that do not favor a direction of information flow are classified as neutral.

### Senders and receivers of the human connectome

Whole-brain white matter tractography was applied to high-resolution diffusion MRI data acquired for *K* = 200 healthy adults (age 21–36, 48.5% female) participating in the Human Connectome Project^30^. Structural brain networks were mapped at several spatial resolutions (*N* = 256,360,512 regions; Methods section, Connectivity data. For each individual, the resulting weighted adjacency matrix was thresholded at 10%, 15%, and 20% connection density to eliminate potentially spurious connections^31^, and subsequent analyses were carried out on the obtained weighted connectomes. Communication matrices quantifying the communication efficiency between every pair of regions were computed (Fig. 2a, Methods section, Network communication models) and used to derive measures of send-receive communication asymmetry (Methods section, Communication asymmetry test). We focus on describing the results for *N* = 360, corresponding to a state-of-the-art cortical atlas derived from high-quality multi-modal data from the HCP^32^, and 15% connection density. Results for other connection densities and parcellation resolutions can be found in the Supplementary Information.

**Fig. 3:**
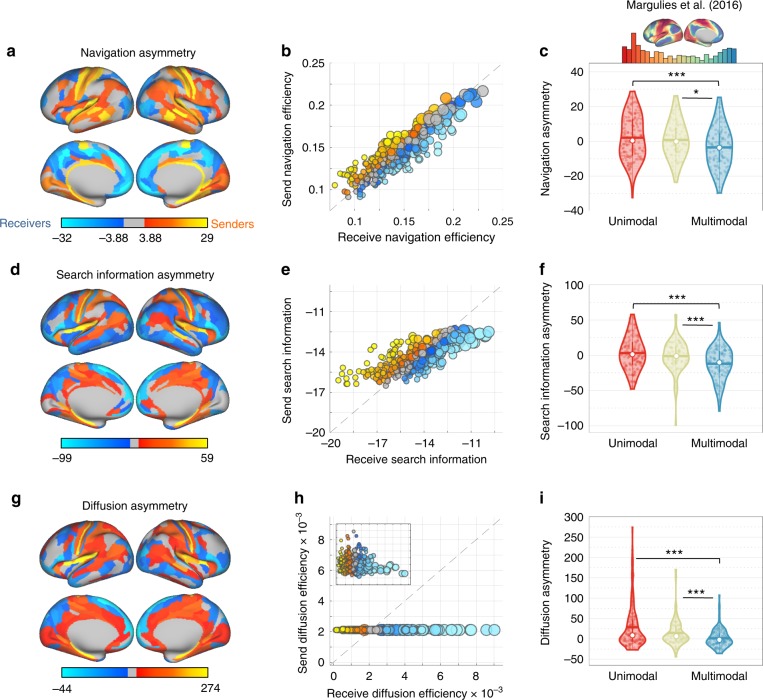
Send-receive communication asymmetry in the human connectome (*N* = 360 at 15% connection density). **a** Cortical projection of send-receive asymmetry values under navigation. Regions associated with significant send-receive asymmetry are classified as putative senders (orange) and receivers (blue). Regions colored gray are neutral and do not show significant send-receive asymmetry. **b** Scatter plot showing correlation between send (vertical axis) and receive (horizontal axis) efficiency across regions under navigation. Send and receive efficiency values were aggregated across all individuals for each region. Markers are colored according to send-receive asymmetry values (colors approximately match that of panel **a**). Small, medium and large markers represent nodes with low (*κ* < 50), medium (50 ≤ *κ* ≤ 100) and high (*κ* > 100) degree, respectively. The dashed line marks the *x* = *y* identity line. The distance between markers and the identity line provides a geometric interpretation of regional bias towards sending (*x* < *y*) or receiving (*x* > *y*) efficiencies. **c** Top: Distribution of the cortical gradient eigenvectors used as a measure of functional heterogeneity^35^. Bottom: Violin plots showing distribution of send-receive asymmetries for regions classified as unimodal (red), transitional, (beige) and multimodal (blue) regions. Horizontal bars and white circles denote, respectively, the mean and median of the distributions. Stars denote significant differences in between-group medians given by a two-sided Wilcoxon rank sum test (one, two and three stars denote *P* < 0.05, 0.005, 0.0005, respectively). **d**, **e** Search information equivalent of **a**–**c**. (**g**–**i**) Diffusion efficiency equivalent of **a**–**c**

Significant asymmetries in the efficiency of sending versus receiving information were evident for most cortical regions (Fig. 3a, d, g). Regional values of send-receive asymmetry were significantly correlated across regions among the communication measures investigated (navigation and diffusion: *r* = 0.29, navigation and search information: *r* = 0.31, diffusion and search information: *r* = 0.85; all *P* < 10^−7^). Based on these send-receive asymmetries, we classified all regions as senders, receivers or neutral. As expected from the strong correlation between them, diffusion and search information asymmetries led to similar classifications, likely due to their mutual dependence on random walk processes. While communication under navigation is guided by different mechanisms, classification consistency across measures was greater than expected by chance (Supplementary Note 1).

Primary sensory and motor regions were identified as senders (A1, S1, and M1 across all communication measures and V1 for navigation and diffusion). This is consistent with the notion that early auditory, visual and sensory-motor areas constitute the three main input streams to the cortex, being the first cortical regions to process sensory stimuli transmitted from subcortical structures^32,33^. In contrast, expanses of the orbital and polar frontal cortices, the medial and dorsolateral prefrontal cortices, and the precuneus were classified as receivers. These regions have been proposed as putative functional hubs, supporting abstract and high-order cognitive processes by integrating multiple modalities of information^34–36^. Other regions consistently identified as senders included portions of the superior temporal, medial temporal and posterior cingulate cortices, while parts of intraparietal sulcus cortex, dorsal, and ventral streams consistently ranked amongst receivers. The MT+ complex was also a prominent receiver, potentially reflecting the role of higher order sensory regions as integrators of signals transmitted from primary/earlier cortices. Certain regions were classified as senders under one communication measure but receivers under another measure, possibly reflecting how the three communication measures uniquely interact with connectome topology. Inconsistently classified regions included portions of the paracentral, cingulate, middle temporal, and inferior temporal cortices. Details on how to access complete send-receive asymmetry tables and cortical maps are provided in Methods section and Data availability.

Despite significant asymmetries in the efficiency of sending versus receiving information *within* individual regions, these send-receive asymmetries were superposed atop a strong correlation *across* regions between send and receive efficiency (navigation: *r* = 0.95, search information: *r* = 0.79; both *P* < 10^−15^; Fig. 3b, e). In other words, efficient senders were also typically efficient receivers, and vice versa. Therefore, while all senders were by definition significantly more efficient at sending than receiving, some senders were in fact *less* efficient at sending than some receivers. In contrast, send and receive efficiencies were not correlated under diffusion (*r* = −0.1, *P* = 0.1). In addition, send efficiency was relatively uniform across regions under diffusion, while receive efficiency showed markedly higher regional diversity.

Node degree and participation (Supplementary Note 2) were associated with send-receive asymmetries under diffusion and search information (degree: *r* = −0.54, −0.70, participation: *r* = −0.29, −0.32, respectively. All *P* < 10^−7^), with senders and receivers characterized by low and high degree/participation, respectively. We regressed out the influence of degree on send-receive asymmetry and found that primary cortices remained senders while frontal and prefrontal regions remained receivers. We also noticed that sensory-motor and auditory cortices displayed a significantly higher propensity towards outgoing communication than expected based on their degree alone (Supplementary Fig. 2). Send-receive asymmetry under navigation was not correlated with degree or participation (both *P* > 0.05), with senders and receivers uniformly distributed across the degree distribution.

### Senders and receivers situated within cortical gradients

Next, we aimed to test whether regional variation in send-receive asymmetry would accord with established hierarchies of cortical organization^37^. To this end, we investigated whether senders and receivers would reside at opposing ends of a previously delineated unimodal to multimodal gradient of functional connectivity^35^. Under all three communication measures, senders were more likely to be located at the unimodal end of the gradient, whereas the multimodal end was occupied by receivers. More specifically, send-receive asymmetry and the cortical gradient were significantly correlated across regions (*r* = −0.20, −0.30, −0.29, for navigation, diffusion and search information, respectively. All *P* < 10^−4^). These associations remained significant after accounting for the influence of degree on send-receive asymmetries (all *P* < 2 × 10^−4^).

In further analyses, regions were classified as unimodal (U), transitional (T), or multimodal (M) based only on the cortical gradient (Methods section, Cortical gradient of functional heterogeneity). We compared the send-receive asymmetry of these groups and found that unimodal and transitional areas were significantly more efficient at outgoing communication compared to multimodal areas (Fig. 3c, f, i; *P*_*T* > *M*_ = 0.01, 2 × 10^−4^, 2 × 10^−4^ and *P*_*U* > *M*_ = 4 × 10^−4^, 3 × 10^−6^, 8 × 10^−7^, for navigation, search information and diffusion, respectively). Send-receive asymmetry did not differ between unimodal and transitional regions.

These results were generally robust to variations in cortical parcellation and connection density thresholds (Supplementary Figs. 3 and 4). Taken together, our findings demonstrate that decentralized communication measures applied to the undirected human connectome unveil regional distinctions between putative senders and receivers. Furthermore, we show that knowledge about the direction of information flow can elucidate novel organizational structure within established cortical hierarchies, such as the biases towards outgoing and incoming communication efficiency of unimodal and multimodal regions, respectively.

### Senders and receivers of cortical subsystems

Next, we sought to investigate pairwise send-receive asymmetries between large-scale cortical subsystems (Supplementary Fig. 5). We assigned cortical regions to distributed cognitive systems according to established resting-state networks comprising *M* = 7 and 17 subsystems^38^. In addition, we employed a multimodal partition of the cortex into *M* = 22 spatially contiguous subsystems^32^. Regional communication matrices were downsampled to subsystem resolution and send-receive asymmetries were computed at individual and pairwise subsystem levels (Methods section, Cortical subsystems).

**Fig. 4:**
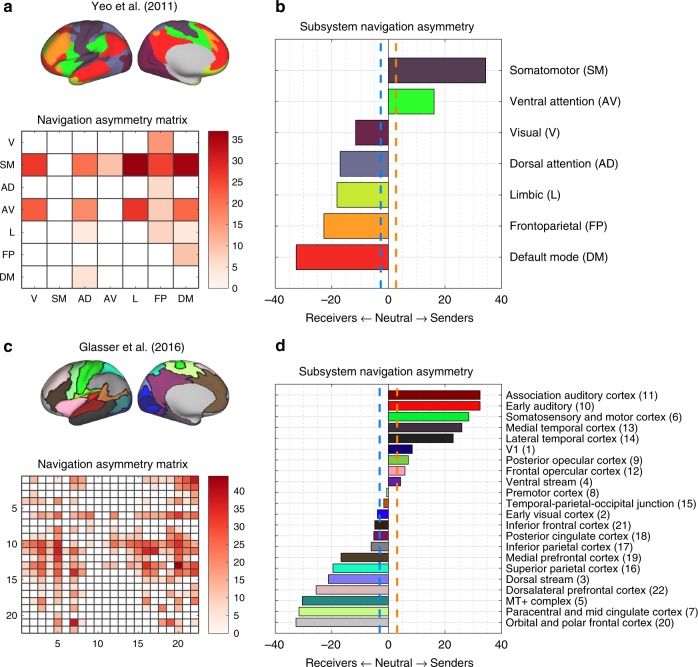
Send-receive asymmetry of cortical subsystems under navigation (*N* = 360 at 15% connection density). **a** Projection of *M* = 7 distributed resting-state networks onto the cortical surface (top) and send-receive asymmetry matrix under navigation (bottom). A matrix element *A*(*i*, *j*) > 0 denotes that communication occurs more efficiently from *i* to *j* than from *j* to *i*. Send-receive asymmetry values that did not survive multiple comparison correction were suppressed and appear as white cells. For ease of visualization and without loss of information (since *A*(*i*, *j*) = −*A*(*j*, *i*)), negative values were omitted. **b** Resting-state networks ranked by propensity to send (top) or receive (bottom) information. Dashed vertical lines indicate a significant bias towards outgoing (orange) and incoming (blue) communication efficiency. **c**, **d** Same as **a**, **b**, but for *M* = 22 spatially contiguous cortical subsystems. Numbers listed next to module names identify corresponding rows and columns in the asymmetry matrix

In keeping with the regional findings, pairwise send-receive asymmetry values were significantly correlated across the communication measures investigated (e.g., navigation and diffusion: *r* = 0.60, navigation and search information: *r* = 0.66, diffusion and search information: *r* = 0.96; all *P* < 10^−15^, *M* = 17). Given the consistency of findings across communication measures, we focus on navigation in this section (Fig. 4). Complete results for navigation, diffusion and search information are shown, respectively, in Supplementary Figs. 6–8).

As shown in Fig. 4a, b, the somatomotor and ventral attention networks were the most prominent senders for the *M* = 7 partition. Prominent receivers included the default mode, frontoparietal and limbic networks, which were more efficiently navigated from a number of cognitive systems than vice versa. Interestingly, adopting a higher resolution functional partition (*M* = 17) suggested that sub-components of coarse (*M* = 7) resting-state networks can assume different roles as senders and receivers. For instance, the visual network was segregated into early (e.g., V1 and V2) and late areas of the visual cortex (e.g., MT+ complex and dorsal and ventral streams), with the first being a sender and the latter a receiver (Supplementary Fig. 6). Other systems that exhibited this behavior included the ventral attention, limbic, somatomotor and default mode networks. These findings reiterate that, despite the presence of asymmetries in send-receive efficiency, cognitive systems are not exclusively capable of sending or receiving, suggesting connectome topology may allow for context-dependent directionality of neural information flow between functional networks.

We also identified senders and receivers for a high-resolution cortical partition comprising *M* = 22 subsystem^32^. This enabled a fine-grained, yet visually interpretable, characterization of send-receive asymmetries (Fig. 4c). Cortical domains associated with auditory, somatosensory and motor processes ranked amongst the strongest senders, while frontal and prefrontal areas consistently featured amongst the most prominent receivers (Supplementary Figs. 6–9). Together, these results provide putative multi-scale maps of how the structural substrate of the human connectome may facilitate directional information flow between cognitive subsystems.

### Senders, receivers, and effective connectivity

We sought to validate our characterization of subsystems as senders or receivers using an independent data modality. To this end, time series summarizing the functional dynamics of cortical subsystems were extracted from resting-state functional MRI data for the same *K* = 200 HCP participants. For each individual, we used spectral DCM^39,40^ to compute effective connectivity between cortical subsystems (*M* = 7,17,22, see Methods section, Effective connectivity). Pairwise effective connectivity asymmetry was computed at the scale of subsystems by applying the previously described asymmetry test to the estimated effective connectivity matrices (Fig. 2c). Importantly, effective connectivity is an inherently directed (asymmetric) measure of connectivity. This allowed us to test whether send-receive asymmetries in communication efficiency (derived from diffusion MRI) and effective connectivity (derived from resting-state fMRI) are correlated (Fig. 2d).

**Fig. 5:**
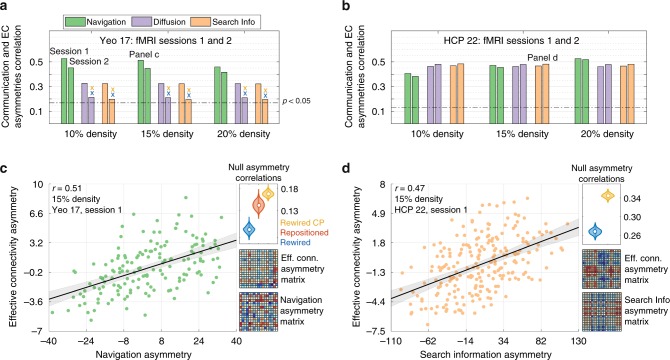
Relationship between send-receive asymmetry and directionality of effective connectivity (*N* = 360). **a** Bars denote the Pearson correlation coefficients between send-receive and effective connectivity asymmetries for *M* = 17 cortical subsystems. Bars are colored according to the three communication measures: (i) navigation (green), (ii) diffusion (violet), and (iii) search information (beige). Correlations were computed for two independent resting-state fMRI sessions (Sessions 1 and 2) and multiple structural connection density thresholds (10, 15, and 20%). Significance threshold of *P* < 0.05 is indicated with a dotted line. Crosses mark associations that were *not* statistically stronger than those found in families of 1000 rewired (blue) and 1000 cost-preserving rewired (yellow) connectomes, respectively (repositioned connectomes, relevant for navigation, led to statistically weaker associations in all scenarios). **b** Replication of Panel **a** for a cortical partition comprising *M* = 22 subsystems. **c** Left: scatter plot illustrating the correlation between navigation and effective connectivity asymmetries for *M* = 17, 15% connection density and fMRI session 1. Shadows denote the 95% bootstrapped confidence interval. Top-right: Distribution of correlations obtained for families of 1000 rewired (blue), repositioned (red) and cost-preserving rewired (yellow) connectomes. Bottom-right: Send-receive asymmetry matrices for effective connectivity (resting-state functional MRI) and navigation (diffusion MRI). The upper-triangular elements of these two matrices were correlated to test whether senders and receivers were consistently identified across independent modalities. **d** Replication of Panel **c** for search information, *M* = 22, 10% connection density and fMRI session 2. EC effective connectivity, *r* Pearson correlation coefficient

Communication and effective connectivity send-receive asymmetries were significantly correlated across pairs of subsystems (Fig. 5). These associations were significant for all three communication measures and were replicated across two independent resting-state functional MRI sessions and multiple structural connection densities. For instance, for *M* = 17, fMRI session 1 and 15% connection density, we found *r* = 0.51, 0.32, 0.32 for navigation, diffusion and search information, respectively (all *P* < 10^−4^). Similarly, for *M* = 22, fMRI session 2 and 15% connection density, we obtained *r* = 0.45, 0.48, 0.48 for navigation, diffusion and search information, respectively (all *P* < 10^−12^). No significant correlations were found for *M* = 7, possibly due to the lack of statistical power afforded by only 21 data points comprising the upper triangle of asymmetry matrices. These results suggest that biases in the directionality of neural signaling inferred from the structural connectome are related to the directions of causal functional modulation during rest. Therefore, they establish a correspondence between structural (connectome topology and network communication measures) and functional (effective) directions of neural information flow.

We sought to determine whether the above association between communication and effective connectivity could be explained by certain properties of connectome organization. We generated ensembles of randomized connectomes in which (i) connectome topology was rewired while preserving degree distribution^41^; (ii) connectome topology was rewired while preserving degree distribution and total network cost (defined as the sum of Euclidean distances between structurally connected nodes^14^); and (iii) nodes were spatially repositioned while preserving topology (relevant only for navigation; see Supplementary Note 3). For all families of randomized connectomes, correlations between asymmetries in effective connectivity and communication efficiency were significantly decreased compared to empirical results (e.g., Fig. 5c, d top-right corner, all *P* < 10^−3^; with the exception of diffusion and search information for the *M* = 17 partition in fMRI session 2, Fig. 5a). These results indicate that the relationship between send-receive asymmetry and directionality of effective connectivity cannot be explained by the combination of connectome degree distribution and network cost, since these properties were preserved in random ensembles (i) and (ii). For navigation, random ensemble (iii) highlights the importance of connectome geometry in addition to topology.

### Control analyses

Having established several properties of the senders and receivers of the human connectome, we aimed to determine whether our results were robust to alternative definitions of send-receive communication asymmetry and changes in our connectome mapping pipeline. First, we redefined our communication asymmetry measure using non-parametric Wilcoxon rank sum tests instead of *t*-tests (Supplementary Note 4). This approach ensures that send-receive asymmetries are robust to deviations from normality and outliers. Second, we computed the send-receive asymmetries of connectomes derived with probabilistic tractography (Supplementary Note 5). Third, we investigated send-receive asymmetries in connectomes including subcortical structures (Supplementary Note 6). Send-receive asymmetries were compatible across all three scenarios and remained consistently associated with the directionality of effective connectivity (Supplementary Fig. 10). Interestingly, while the classification of nodes into senders and receivers in connectomes containing subcortical structures remained largely unaltered, biases towards incoming or outgoing communication were less pronounced (Supplementary Figs. 12 and 13), suggesting a role of subcortical structures as mediators of neural signaling directionality.

### Senders and receivers of non-human connectomes

The association between effective connectivity directionality and the send-receive asymmetry of undirected connectomes indicates that signaling directions in the human brain are not exclusively determined by axonal directions. To further quantify this observation, we next sought to establish the extent to which signaling directionality is determined by axonal directions per se, compared to other potential determining factors such as network topology and geometry.

Invasive connectome reconstruction techniques allow for the resolution of axonal directionality, producing directed connectomes for a host of non-human species^42^. Here, we consider the connectomes of the fruit fly (*Drosophila*)^43,44^, mouse^45,46^ and macaque^47^ (Methods section, Non-human connectomes). We began by computing the send-receive asymmetries of these directed connectomes. In this case, communication asymmetry is introduced both by the asymmetric character of the network communication measures and by the presence of directed connections. Next, we symmetrized the connectomes by removing connection directionality, so that all connections could be traversed bidirectionally (Methods section, Symmetrized non-human connectomes), and recomputed send-receive asymmetries for the resulting undirected networks. In this scenario, as with human undirected connectomes, asymmetries are introduced solely by the asymmetry inherent to the network communication measures. We tested whether send-receive asymmetry values computed in the directed (original) and undirected (symmetrized) non-human connectomes were correlated across regions. Evidence of a correlation would suggest that the undirected topology and geometry of connectomes are influential in determining the directionality of neural signaling in the absence of directed connections.

**Fig. 6:**
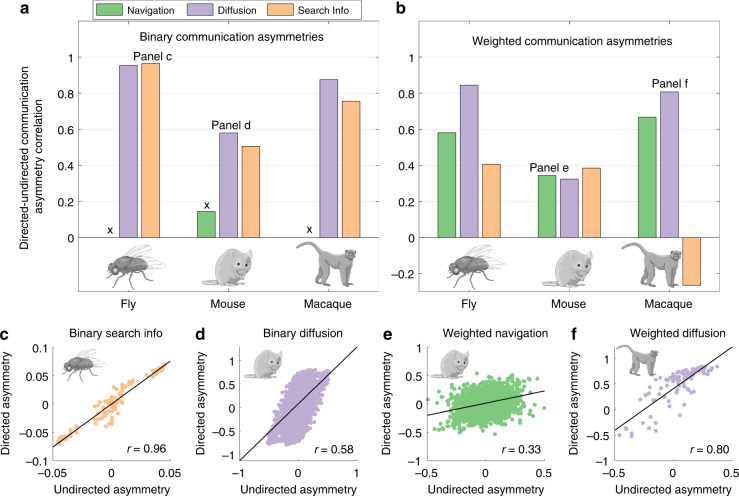
Directed and undirected (symmetrized) send-receive asymmetries in non-human connectomes. **a** Pearson correlation coefficient across regions in send-receive asymmetry values between directed and undirected navigation (green), diffusion (violet) and search information (beige) asymmetries for binarized (unweighted) connectomes. Black crosses indicate *non-significant* (*P* > 0.05) or undefined (in the case of lack of communication asymmetry) correlations. **b** Same as panel **a**, but for weighted connectomes. **c**–**f** Scatter plots illustrating the association between directed and undirected communication asymmetries (*r*: Pearson correlation coefficient, *P*: associated *P*-value)

We found that undirected and directed send-receive asymmetries were correlated for binarized (fly: *r* = 0.95, 0.96, mouse: *r* = 0.58, 0.50, macaque: *r* = 0.87, 0.75, for diffusion and search information asymmetries, respectively; Fig. 6a, c, d) and weighted (fly: *r* = 0.58, 0.84, 0.41, mouse: *r* = 0.34, 0.32, 0.38, macaque: *r* = 0.67, 0.80, −0.26, for navigation, diffusion and search information asymmetries, respectively; Fig. 6b, e, f) non-human connectomes. All reported *r* had *P* < 10^−10^ and thus survived Bonferroni correction for multiple comparisons. The exception was the association for the macaque weighted search information (*P* = 0.01), potentially indicating the spurious nature of this negative correlation. It is worth noting that binary navigation paths are seldom asymmetric for densely connected networks such as non-human connectomes, resulting in weak/undefined correlations between directed and undirected send-receive asymmetries. In addition, due to the presence of connection weight asymmetries between bidirectionally connected node pairs, original and symmetrized connectomes are more similar for unweighted than weighted networks, explaining the stronger associations observed for binarized connectomes.

Finally, we observed that regional senders and receivers of undirected (symmetrized) non-human connectomes also recapitulated putative hierarchies of functional specialization (Supplementary Note 7). For instance, macaque sensory, visual and motor areas were senders, while portions of the frontal and prefrontal cortices were receivers (Supplementary Figs. 14g, h, i and 15). Collectively, these findings provide further evidence that the directionality of neural signaling is partially determined by the undirected architecture of nervous systems across species.

## Discussion

The present study focused on characterizing the directionality of neural information flow arising from the application of decentralized network communication measures to connectomes. In a recent study, Avena-Koenigsberger and colleagues presented a first account of differences between send and receive communication in brain networks^16^. Here, we build on these efforts by contributing a statistical framework to compute send-receive communication asymmetry. We apply this framework to identify putative sender and receiver brain regions, as well as pairwise maps of neural signaling directionality for the nervous systems of several species.

Send-receive asymmetry recapitulated hierarchical patterns of cortical organization from a structural connectivity standpoint. Several studies of axonal tract-tracing and non-human connectomes^34,48,49^, macroscale gradients of cortical organization^35,37,50^, and computational models of neuronal dynamics^27,51–54^ converge to a common conceptualization of a cortical hierarchy of functional specialization. The bottom of the hierarchy tends to comprise high-frequency, low-degree, unimodal, sensory and motor areas that constitute the main inputs of perceptual information to the brain. At the top, low-frequency, high-degree, multimodal regions are conjectured to integrate multiple streams of information in order to support higher cognitive functions. Our observations of a send-receive spectrum of cortical regions and subsystems complements this description of neural organization, placing senders and receivers, respectively, at the unimodal and multimodal ends of the hierarchy.

Previous studies have demonstrated that navigation efficiency, search information and diffusion computed on structural connectomes are capable of inferring resting-state functional connectivity^8,15,24^. Here, we provided further evidence for the utility of decentralized communication models by showing an association between send-receive asymmetry—inferred from connectomes mapped with tractography and diffusion MRI—and directionality of effective connectivity—computed from spectral DCM applied to resting-state fMRI. This relationship was robust to variations in tractography algorithms, cortical subsystem parcellations, treatment of subcortical structures, send-receive asymmetry statistical tests, structural connection density thresholds and two independent resting-state fMRI sessions. Compared to the first fMRI session, the strength of this relationship was weaker in the second session for the case of the *M* = 17 cortical subsystems. This may be due to the effect of MRI phase-encoding differences between the two sessions (Methods section, Send-receive effective connectivity asymmetry) on particular subsystem parcellations, although this requires further investigation.

Recent work has demonstrated the validity of spectral DCM in multi-site longitudinal settings^55^ and using optogenetics combined with functional MRI in mice^56^. The use of spectral DCM instead of the traditional task-based DCM was motivated by two important factors. First, spectral DCM infers effective connectivity from resting-state fMRI data, allowing validation of our findings independent of hypotheses about the directionality of causal connectivity specific to certain task scenarios. In addition, recent evidence indicates that functional connectivity topology at rest shapes task-evoked fluctuations, highlighting the cognitive relevance of resting-state neural dynamics^57,58^. Second, spectral DCM is capable of handling relatively large networks comprising many regions^59^. This enabled a direct comparison between asymmetries in send-receive efficiency and effective connectivity at the level of subsystems spanning the whole cerebral cortex. Our results provide cross-modal evidence that network communication measures accurately capture aspects of directional causal influences between neural systems. Structurally derived communication asymmetry may help formulate hypotheses for DCM studies, potentially reducing the search space of candidate network models^60^. In addition, send-receive asymmetry may be useful in understanding asymmetric responses in functional dynamics following exogenous stimulation of brain regions^27,52^.

The analyses of human undirected connectomes indicate that meaningful patterns of neural signaling directionality can be inferred without knowledge of the directions of axonal projections. We provided further evidence for this notion by examining non-human connectomes, for which information on axonal directionality is invasively derived. Send-receive asymmetries computed for directed connectomes were significantly associated to those derived from networks for which the directionality of connections was suppressed. Moreover, senders and receivers computed from undirected version of non-human connectomes also recapitulated putative functional roles of brain regions. These results indicate that despite the documented importance of directed connections^47,61^, the undirected architecture of nervous systems also imposes constraints on signaling directionality. This may suggest the presence of fundamental, cross-species organization properties of brain networks that facilitate decentralized communication between neural elements. It is worth noting that send-receive asymmetry is more pronounced between pairs of regions that are not directly connected, for which communication takes place along multi-hop paths. Consequently, as formulated here, send-receive asymmetry is not well suited to perform inference for structurally connected nodes, and thus should not be conceptualized as a methodology to transform an undirected structural connectome into a directed graph. Future work exploring alternative formulations of communication asymmetry could attempt to infer directed structural traits from undirected connectomes.

Send-receive asymmetry is a result of the interaction between asymmetric network communication models and the topology and geometry of brain networks. Navigation depends on local knowledge of the network’s spatial embedding to identify communication paths, while diffusive processes rely solely on local connectivity knowledge to propagate signals. Despite these conceptual differences, navigation efficiency, search information and diffusion efficiency led to similar patterns of send-receive asymmetry. The classification of cortical regions into senders and receivers, as well as the association with effective connectivity directionality was generally consistent across measures. This indicates that our results may be primarily driven by how the architecture of brain networks gives rise to general patterns of communication asymmetry, rather than by specific strategies of neural signaling. An interesting deviation from these consistencies was observed in the relationship between regional send and receive efficiencies. While navigation and search information showed a positive correlation between send and receive efficiencies, this was not the case for diffusion. Moreover, as previously reported^16^, diffusion receive efficiency showed markedly greater regional variation compared to diffusion send efficiency. This is a consequence of high degree nodes being more accessible to incoming random walkers than low degree ones.

We reiterate that a significant send-receive asymmetry does not preclude information transfer in a particular direction, in the same way that regions classified as senders (receivers) are capable of receiving (sending) information. Interestingly, we also found that coarse functional networks with significant biases towards incoming or outgoing communication are typically comprised of subcomponents placed along different positions of the sender-receive spectrum. This may facilitate feedback loops in which high-order regions send information to sensory cortices, allowing for flexible and context-dependent transfer of neural information. These results support the notion that cortical computations do not follow a strictly serial paradigm, but rather involve distributed hierarchies of parallel information processing^38,48^.

Several limitations of the present study should be considered. Send-receive asymmetry was defined statistically across subjects. Future developments are necessary to conceptualize robust measures of subject-level send-receive asymmetry. In addition, alternative asymmetric network communication measures such as Markovian queuing networks^62^, linear transmission models of spreading dynamics^19,20^ and cooperative learning^63^ can lead to further insight into the large-scale directionality of neural signaling. Additional measures of directed functional connectivity such as transfer entropy and Granger causality may offer supplementary cross-modal validation of send-receive asymmetry. Importantly, tractography algorithms are prone to known biases, potentially influencing results regarding human structural connectomes^31,64,65^. Lastly, navigation was computed based on the Euclidean distance between brain regions. Alternative distance measures taking it account axonal fiber length may provide more biologically realistic guidance for connectome navigation.

In conclusion, we showed that the large-scale directionality of neural signaling can be inferred, to a significant extent, from the interaction between decentralized network communication measures and the undirected topology and geometry of brain networks. These results challenge the belief that connectomes mapped from in vivo diffusion data are unable to characterize asymmetric interactions between cortical elements. Our findings introduce decentralized network communication models as a new avenue to explore directional functional dynamics in human and non-human connectomes.

## Methods

### Connectivity data

Minimally preprocessed diffusion-weighted MRI data from 200 healthy adults (age 21–36, 48.5% female) was obtained from the Human Connectome Project (HCP)^30^. Details about the diffusion MRI acquisition and preprocessing are described in^66,67^.

Connectome analyses are sensitive to the number of nodes used to reconstruct brain networks^68^. We aimed to reproduce our key findings for human connectomes constructed with different granularities of cortical segmentation comprising *N* = 256,360,512 regions/nodes. The parcellations for *N* = 256,512 segment the cortex into approximately evenly sized regions that respect predefined anatomical boundaries. Details on the construction of these parcellations are described in^15^. In addition, we mapped connectomes using the HCP MMP1.0 atlas (*N* = 360), a cortical parcellation based on multi-modal data from the HCP^32^.

Diffusion tensor imaging combined with a deterministic tractography pipeline was used to map connectomes for each individual. Deterministic tractography leads to less false positive connections than other reconstruction methods, and thus may better suit connectome mapping compared to alternative tractography methods^31,64,65^. Computations were carried out using MRtrix3^69^ with the following parameters: FACT tracking algorithm, 5 × 10^6^ streamlines, 0.5 mm tracking step-size, 400 mm maximum streamline length and 0.1 FA cutoff for termination of tracks. Connection strength between a pair of regions was determined as the number of streamlines with extremities located in the regions divided by the product of the surface area of the region pair, resulting in a *N* × *N* weighted connectivity matrix per subject. For each individual, the resulting weighted adjacency matrix was thresholded at 10, 15, and 20% connection density to eliminate potentially spurious connections^31^, and subsequent analyses were carried out on the obtained weighted connectomes.

The fruit fly connectome was mapped using images of 12,995 projection neurons in the female *Drosophila* brain available in the FlyCircuit database^43,44^. Single neurons were labeled with green fluorescent protein and traced from whole brain three-dimensional images. Individual neurons were grouped into 49 local processing units with specific morphology and function. The resulting connectome is a 49 × 49 weighted, directed, whole-brain network for the fruit fly, with 83% connection density.

The Allen Institute for Brain Science mapped the mesoscale topology of the mouse nervous system by means of anterograde axonal injections of a viral tracer^45^. Using two-photon tomography, they identified axonal projections from the 469 injections sites to 295 target regions. Building on these efforts, Rubinov and colleagues constructed a directed, bilaterally symmetric, whole-brain network for the mouse, comprising *N* = 112 cortical and subcortical regions with 53% connection density^46^. Connections represent interregional axonal projections and their weights were determined as the proportion of tracer density found in target and injected regions.

Markov and colleagues applied 1615 retrograde tracer injections to 29 of the 91 areas of the macaque cerebral cortex, spanning occipital, temporal, parietal, frontal, prefrontal and limbic regions^47,70^. This resulted in a 29 × 29 weighted, directed, interregional sub-network of the macaque cortico-cortical connections with 66% connection density. Connection weights were estimated based on the number of neurons labeled by the tracer found in source and target regions, relative to the amount found in whole brain.

### Network communication measures

A weighted connectome can be expressed as a matrix *W* ∈ ℝ ^*N* × *N*^, where *W*_*ij*_ is the connection weight between nodes *i* and *j*. Connection weights are a measure of similarity or affinity, denoting the strength of the relationship between two nodes (e.g., streamline counts in tractography or fraction of labeled neurons in tract tracing). The computation of communication path lengths mandates a remapping of connection weights into lengths, where connection lengths are a measure of the signaling cost between two nodes^11^. The transformation *L* = −log_10_(*W*/(*max*(*W*) + *min*(*W*_>0_)) ensures a monotonic weight-to-length remapping that attenuates extreme weights^8,71^, where *min*(*W*_>0_) denotes the smallest positive value in W, preventing the remapping of the maximum value of *W* to 0.

Navigation (also referred to as greedy routing) is a decentralized network communication model that utilizes information about the network’s spatial embedding to route signals without global knowledge of network topology^28^. Navigation is reported to achieve near-optimal communication efficiency in a range of real-world complex networks, including the connectomes of several species^15,17,18^.

Navigation from node *i* to *j* was implemented as follows. Progress to *i*’s neighbor that is closest in distance to *j*. Repeat this process for each new node until *j* is reached—constituting a successful navigation path—or a node is revisited—constituting a failed navigation path. The distance between two nodes was computed as the Euclidean distance between the centroids of their respective gray matter regions. For each parcellation resolution, a single Euclidean distance matrix was computed in standard space and utilized to guide the navigation of each individual’s connectome.

Let Λ denote the matrix of navigation path lengths. If node *i* cannot navigate to node *j*, Λ_*ij*_ = ∞. Otherwise, Λ_*ij*_ = *L*_*iu*_ + … + *L*_*vj*_, where {*u*, …, *v*} is the sequence of nodes visited during navigation. Note that while navigation paths are identified based on the Euclidean distance between nodes, navigation path lengths are computed in terms of connection lengths derived from the structural connectivity matrix *W*. Navigation efficiency is given by *E*_nav_(*i*, *j*) = 1/Λ_*ij*_, where *E*_nav_(*i*, *j*) is the efficiency of the navigation path from node *i* to *j*^15^.

A diffusion process is a network communication model whereby information is broadcast along multiple paths simultaneously^22^. Diffusion can be understood in terms of agents, often termed random walkers, which are initiated from a given region and traverse the network independently of each other by randomly selecting a connection to follow out from each successive region that is visited. Diffusive communication does not mandate assumptions on global knowledge of network topology, constituting, from this perspective, a biologically plausible model for neural communication^9^. Diffusion efficiency^23^ is related to how many intermediate regions (synapses), on average, a naive random walker needs to traverse to reach a desired destination region.

Let *T* denote the transition probability matrix of a Markov chain process with states corresponding to nodes in the adjacency matrix *W*. The probability of a random walker at node *i* stepping to node *j* is given by $T_{ij} = W_{ij}/\mathop {\sum}\nolimits_{n = 1}^N {W_{in}}$. The expected number of hops 〈*H*_*ij*_〉 a random walker takes to travel from node *i* to node *j* is given by^72^:

1 $$\left\langle {H_{ij}} \right\rangle = \mathop {\sum}\limits_{h = 0}^\infty {hP(H_{ij} = h)} = \mathop {\sum}\limits_{h = 0}^\infty {P(H_{ij} > h)} .$$

This result is given by the fact that the expected value of a random variable is given by the sum of its complementary cumulative distribution. The probability of a walker requiring more than *h* hops to reach node *j* is equal to the sum of the probabilities of the walker being at any node other than *j* after exactly *h* hops. To compute this, we define *T*_*j*_ as the matrix *T* with all elements in the *j*th column set to zero, so that it is impossible for a walker to arrive at node *j*. This way, we have $P(H_{ij} > h) = \mathop {\sum}\nolimits_{n = 1}^N {\left[ {T_j^h} \right]_{in}}$, where $\left[ {T_j^h} \right]_{in}$ expresses the probabilities of walkers departing from *i* and reaching any other node expect *j* in exactly *h* hops. It follows that

2 $$\left\langle {H_{ij}} \right\rangle = \mathop {\sum}\limits_{h = 0}^\infty {\mathop {\sum}\limits_{n = 1}^N {\left[ {T_j^h} \right]_{in}} } = \mathop {\sum}\limits_{n = 1}^N {\left[ {\left( {1 - T_j} \right)^{ - 1}} \right]_{in}} ,$$

with the last derivation step following from the summation of an infinite geometric sequence. Further details on this derivation can be found in refs. ^11,23,72^. The diffusion efficiency communication matrix is given by *E*_*dif*_ (*i*, *j*) = 1/*H*_*ij*_, where *E*_*dif*_ (*i*, *j*) quantifies the efficiency of information flow from node *i* to node *j* under a diffusive process^23^.

Search information relates to the probability that a random walker will serendipitously travel between two nodes via their shortest path^29^, quantifying the extent to which efficient routes are hidden in the network topology. Previous studies suggest node pairs with an accessible shortest path—characterized by low search information—tend to show stronger resting-state functional connectivity^8^.

The connection length matrix *L* can be used to compute Ω, where Ω_*ij*_ = {*u*, …, *v*} denotes the sequence of nodes traversed along the shortest path from node *i* to node *j*. The search information from *i* to *j* is given by *SI*_*ij*_ = −log_2_(*P*(Ω_*ij*_)), where *P*(Ω_*ij*_) = *T*_*iu*_ + … + *T*_*vj*_ and *T* is the transition probability matrix. We define communication efficiency under search information as *E*_*si*_(*i*, *j*) = −*SI*_*i*,*j*_. This way, *E*_*si*_(*i*, *j*) quantifies the accessibility of the Ω_*ij*_ shortest path under diffusive communication.

### Send-receive communication asymmetry measures

Send-receive communication asymmetry matrices *A* ∈ ℝ ^*N* × *N*^ were computed as detailed in the Results, Measures of send-receive communication asymmetry section. For each pair of regions or subsystems, a one-sample *t*-test was used to assess whether the mean of the send-receive asymmetry values across all individuals was significantly different from 0 (see Supplementary Note 3 for an investigation of normality assumptions involved in *t*-tests and send-receive asymmetries based on non-parametric statistics). Bonferroni correction was then performed to control for the *N*(*N* − 1)/2 multiple comparisons corresponding to distinct pairs of regions. This was repeated for each of the three communication measures.

The communication asymmetry matrix *A* refers to pairwise asymmetric interactions between regions. We performed a similar test to derive a regional (i.e., node-wise) measure of send-receive asymmetry. Let {*S*, *R*} ∈ ℝ ^*N* × *K*^ denote, respectively, the average send and receive efficiencies of nodes in the network such that $S(i,k) = 1/N\mathop {\sum}\nolimits_{j = 1}^N {C(i,j,k)}$ and $R(j,k) = 1/N\mathop {\sum}\nolimits_{i = 1}^N {C(i,j,k)}$. The difference between outgoing and incoming communication efficiencies of node *i* is given by *δ*(*i*, *k*) = *S*(*i*, *k*)−*R*(*i*, *k*). Analogous to the pairwise asymmetry test, we performed a one-sample *t*-test to determine whether the mean of the distribution *δ*(*i*, *k* = 1…*K*) is significantly different to 0. The resultant t-statistic, termed *a*(*i*), quantifies the communication asymmetry of node *i* by taking into account all of its incoming and outgoing communication efficiencies. Nodes with significant and positive (negative) *a* were classified as senders (receivers), while non-significant values of *a* were characterized neutral nodes. For each network communication measure, Bonferroni correction was performed to control for multiple comparisons across the *N* regions.

Regionally aggregated send and receive efficiencies depicted in the scatter plots of Fig. 3e, h were computed as $s(i) = 1/K\mathop {\sum}\nolimits_{k = 1}^K {S(i,k)}$ and $r(j) = 1/K\mathop {\sum}\nolimits_{k = 1}^K {R(j,k)}$, respectively. For navigation (Fig. 3b), we display the median send and receive efficiencies in order to attenuate outlier efficiency values and aid visualization.

Non-human directed connectomes were constructed from the results of numerous invasive experiments, often combining experiments across multiple animals of a given species to yield a single, representative connectome. As a result, non-human brain networks were not available for multiple individuals, precluding use of the communication asymmetry test defined for human connectomes. As an alternative, for non-human brain networks, we computed the communication asymmetry between nodes *i* and *j* as *A*(*i*, *j*) = (*E*(*i*, *j*) − *E*(*j*, *i*))/(*E*(*i*, *j*) + *E*(*j*, *i*)), where *E* is a communication efficiency matrix. While this measure does not constitute a statistical test of communication asymmetry, it allows us to evaluate differences in the directionality of information flow of non-human nervous systems.

### Cortical gradient of functional heterogeneity

Margulies and colleagues applied a diffusion embedding algorithm to resting-state fMRI data to identify latent components describing maximum variance in cortical functional connectivity^35^. The obtained components, termed “gradients”, are conjectured to describe macroscale principles of cortical organization^37^. In particular, the resultant principal gradient (*G*_1_) separated unimodal from multimodal regions, spanning a spectrum from primary sensory-motor areas on one end, to the regions comprising the default-mode network on the other. We used this gradient as a quantitative measure of cortical functional heterogeneity and compared it to regional send-receive communication asymmetries. To this end, we downsampled the gradient from vertex to regional resolution by averaging the values comprising each of the *N* = 256,360,512 cortical areas defined by the parcellations that we used. Regions were grouped into the unimodal (*G*_1_ ≤ −2), transitional (−2 < *G*_1_ < 2) and multimodal (*G*_1_ ≥ 2) groups shown in Fig. 3.

### Cortical subsystems

Yeo and colleagues proposed a widely used partition of the cortical surface into 7 and 17 resting-state functional networks^38^. These networks constitute distributed (i.e., non-contiguous) functional communities that have been implicated in a wide range of cognitive demands, as well as in rest. Glasser and colleagues used multimodal HCP data to identify 360 cortical regions. Subsequently, they grouped these regions into 22 contiguous subsystems based on geographic proximity and functional similarities^32^. We use these definitions of cortical partitions to investigate send-receive communication asymmetry at the level of subsystems.

First, we transformed the Yeo partitions (*M* = 7,17) from vertex to regional resolution. This was achieved by assigning each of *N* = 360 cortical regions to the resting-state network with the largest vertex count within the vertices comprising the region. The HCP partition (*M* = 22) does not necessitate this step, since it is already defined in terms of the *N* = 360 of the Glasser atlas.

Second, we downsampled individual communication efficiency matrices from regional (*N* = 360) to subsystem resolution (*M* = 7,17,22) by averaging the pairwise efficiency of nodes assigned to the same subsystem. For two subsystems *u* and *v*, we have

3 $$E_{uv}^\prime = \frac{1}{{|M_u||M_v|}}\mathop {\sum}\limits_{\scriptstyle i \in M_u\atop\\ \scriptstyle j \in M_v} {E_{ij}} ,$$

where *M*_*u*_ and |*M*_*u*_| denote, respectively, the set and number of regions belonging to subsystem *u*, *E* ∈ ℝ ^*N* × *N*^, and *E*′ ∈ ℝ ^*M* × *M*^. Across *K* subjects, this results in a set of communication matrices *C* ∈ ℝ ^*M* × *M* × *K*^ that is used to compute between-subsystems send-receive communication asymmetries as described in Fig. 2 and in the Results section, Measures of send-receive communication asymmetry.

The send-receive communication asymmetry for individual cortical subsystems was computed analogous to regional communication asymmetries as described in the Methods section, Send-receive communication asymmetry measures. For each network communication measure, Bonferroni correction for *M* and *M*(*M* − 1)/2 multiple comparisons was applied to individual and pairwise subsystems asymmetries, respectively.

### Send-receive effective connectivity asymmetry

Spectral DCM estimates effective connectivity from resting-state fMRI data. It receives as input time series characterizing the functional dynamics of neural activity and a network model describing how these elements are coupled. As opposed to the more common task-based DCM, spectral DCM estimates effective connectivity in the absence of experimental or exogenous inputs, characterizing functional modulations between neural elements based on intrinsic neural fluctuations at rest. Details on the generative models inherent to spectral DCM, as well as the frequency-domain model inversion are described in^39,59^.

Minimally preprocessed resting-state fMRI data for the same *K* = 200 subjects was acquired from the HCP. Functional volumes were acquired during 14m33s at 720 TR, resulting in 1200 time points. Data from two separate sessions (*rfMRI REST1 LR* and *rfMRI REST2 RL*, i.e., right-to-left and left-to-right encoding, performed on different days) was used to compute two estimates of effective connectivity for each subject. HCP acquisition and preprocessing of resting-state fMRI are detailed in^66,67^.

We computed the blood-oxygenation-level-dependent (BOLD) signal of *N* = 360 regions by averaging the time series of all cortical surface vertices belonging to a region. Next, the *N* regions were partitioned into *M* cortical subsystems as described in the Methods section, Cortical subsystems. For each subsystem, we performed a principal component analysis on all the time series belonging to it. The resultant first principal component was used to summarize the functional activity of a subsystem in a single time series. The *M* × 1200 time series of principal components were used as input to spectral DCM, together with a fully connected model of coupling strengths (1_*M* × *M*_), enabling estimation of effective connectivity between subsystems covering the whole cortex^59^. Spectral DCM estimations were carried out using SPM12.

Spectral DCM estimates signed effective connectivity, with positive and negative values indicating excitatory and inhibitory influences, respectively. Under the assumption that both excitatory and inhibitory processes are facilitated by communication between neural elements, we considered the absolute value of the estimated coupling strengths.

The obtained coupling strengths of each subject were concatenated. For each resting-state session, this yielded a *M* × *M* × *K* effective connectivity matrix, which were used to compute effective connectivity asymmetry between cortical subsystems, as described in Fig. 2 and the Results, Measures of send-receive communication asymmetry section.

### Symmetrized non-human connectomes

Directed non-human connectomes (*W*_d_) were symmetrized in order to omit information on axonal directionality. Undirected (symmetric) networks (*W*_u_) were computed as $W_{\rm{u}} = (W_{\rm{d}} + W_{\rm{d}}^{\rm{T}})/2$, ensuring that all original connections in *W*_d_ can be traversed bidirectionally in *W*_u_. Directly connected node pairs do not show send-receive asymmetry under navigation, since both directions of routing will necessarily occur via the single connection linking the two nodes. For this reason, we restricted the analyses in ʻSenders and receivers in non-human connectomesʼ to node pairs that did not share a direct structural connection in *W*_u_. Non-human binary connectomes were constructed by discarding information on connectivity weight and considering only the presence or absence of directed connections. Formally, *B*(*i*, *j*) = 1 if *W*(*i*, *j*) ≠ 0 and *B*(*i*, *j*) = 0 otherwise.

### Reporting summary

Further information on research design is available in the Nature Research Reporting Summary linked to this article.

## Supplementary information

Supplementary Information

Reporting Summary

## Data Availability

All analyses in this study were carried out on publicly available datasets. Structural and effective human brain networks were mapped from Human Connectome Project data^30^ (https://db.humanconnectome.org/). The fruit fly connectome was collated from data available in http://www.flycircuit.tw and can be found in the Supplementary Information of ref. ^44^. The macaque connectome was derived from data available at http://core-nets.org/^47^. The mouse connectome was constructed from resources provided by the Allen Institute for Brain Science (https://mouse.brain-map.org/^45^) and is available in the Supplementary Information of ref. ^46^. The cortical gradient of functional connectivity from ref. ^35^ is available at https://www.neuroconnlab.org/data/index.html. Send-receive communication asymmetry measures, custom MATLAB code, and other data necessary to generate key figures in this work are available at https://github.com/caioseguin/sen_rev.

## Code availability

Functions to compute navigation efficiency, diffusion efficiency and search information are available as part of the Brain Connectivity Toolbox (https://sites.google.com/site/bctnet/). Further analyses and computations were performed using MRtrix3 (www.mrtrix.org/), SPM12 (https://www.fil.ion.ucl.ac.uk/spm/software/spm12/) or custom MATLAB code available at https://github.com/caioseguin/sen_rev.
