# Supplementary material for: Inferring neural signalling directionality from undirected structural connectomes
Source: Nat Commun. 2019 Sep 19;10:4289. doi: 10.1038/s41467-019-12201-w (PMC6753104; doi:10.1038/s41467-019-12201-w)
Supplement: Supplementary file 1 — Supplementary Information [file 41467_2019_12201_MOESM1_ESM.pdf]

Inferring neural signalling directionality from undirected structural connectomes.

Seguin et al., 2019.

Inferring neural signalling directionality from undirected structural connectomes

Caio Seguin[1],* Adeel Razi[2,3,4], and Andrew Zalesky[1,5]
[1]Melbourne Neuropsychiatry Centre, The University of Melbourne and Melbourne Health, Melbourne, VIC 3010, Australia
[2]Monash Institute of Cognitive and Clinical Neurosciences,
and Monash Biomedical Imaging, Monash University, Clayton, Australia
[3]The Wellcome Trust Centre for Neuroimaging, University College London, London, United Kingdom
[4]Department of Electronic Engineering, NED University of Engineering and Technology, Karachi, Pakistan and
[5]Department of Biomedical Engineering, Melbourne School of Engineering,
The University of Melbourne, Melbourne, VIC 3010, Australia
(Dated: August 19, 2019)

SUPPLEMENTARY INFORMATION

Note 1: Consistency of send-neutral-receiver classification of cortical regions across communication measures

Extending our analyses of correlations between send-receive asymmetries across communication measures, we compared the sender-neutral-receiver classifications of cortical regions obtained from navigation efficiency, diffusion efficiency and search information (Supplementary Table I). As expected by their mutual dependency on random walk processes, classifications for send-receive asymmetries under diffusion efficiency and search information were tightly related (76% accuracy, Supplementary Table Ic). While classification under navigation showed less agreement with the other measures (46% and 44% accuracy for diffusion and search information, respectively; Supplementary Table Ia,b), the obtained three-way classification accuracy remained larger than the 33% baseline expected by chance.

We further explored this relationship by considering two-way classifications of regions into sender or not sender, and receiver or not receiver. This allowed us to assess the statistical significance of the association between the classifications from two communication measures by using Fisher's exact test. The significance of the obtained P-values further supports the classification consistency across communication measures (Supplementary Table Id-i).

Note 2: Human connectome modularity and node participation

Modular decompositions were estimated for group-level connectomes computed as the average of all individual connectivity matrices for $N = 256, 360, 512$. The resulting group-level connectomes were thresholded at 10%, 15% and 20% connection density and analysed as weighted networks. Nodes were assigned to modules by means of the Louvain algorithm with iterative fine-tuning [1], as implemented in the Brain Connectivity Toolbox [2]. Briefly, the algorithm aims to identify partitions of the network that maximize within-module connection weights. This notion is formalized by the optimization of the modularity statistic

$$Q = \frac{1}{2m}\sum_{ij}\left[W_{ij} - \gamma\frac{s_i s_j}{2m}\right]\delta_{ij}, \qquad (1)$$

where W_{ij} is the connection weight between nodes i and j, γ is the resolution parameter (set to default value of 1), s_i is the strength of node i, $2m$ is a normalization constant equal to the sum of all connection weights in the network, and $\delta_{ij} = 1$ if i and j are assigned to the same module and 0 otherwise. For each group-level connectome, the Louvain algorithm was applied 100 times and the partition that maximized Q was selected. For each individual run of the algorithm, partitions were fine-tuned for k iterations until $Q_k - Q_{k-1} < 10^{-5}$ or $k = 100$. This procedure resulted in the identification of 7 modules for $N = 256$ (all connection densities); 6 modules for $N = 360$ (all connection densities); and 6, 7, 7 modules for $N = 512$, for 10%, 15%, 20% connection density, respectively.

The participant coefficient measures the diversity of inter-modular connections of individual nodes, and is defined as [3]

$$P_i = 1 - \sum_{g\in G}\left(\frac{s_i(g)}{s_i}\right)^2. \qquad (2)$$

Here, G denotes the set of all modules, s_i the strength of node i and $s_i(g)$ the strength of i within module g. High and low P_i indicate that i has connections to nodes assigned to many and few distinct modules, respectively.

Note 3: Randomized connectomes

We used three families of randomized connectomes: i) topologically randomized (rewired) networks, ii) topologically randomized cost-preserving networks and iii) spatially randomized (repositioned) networks. For K subjects, each individual connectome was used to generate

* caioseguin@gmail.com

ensembles of 1,000 surrogate networks for each family. Computing communication measures for these networks resulted in 1,000 sets of $C_{N\times N\times K}$ null communication efficiency matrices, per family, per communication measure. These sets were downsampled to subsystem resolution and used to compute the correlation between effective connectivity and null network send-receive communication asymmetries. Non-parametric P-values testing the hypothesis of a lack of difference between empirical and null correlations were computed as the proportion of times the communication asymmetry of null networks yielded stronger correlations than the one obtained for the empirical connectome.

Topologically randomized networks were computed using the Maslov-Sneppen rewiring routine [4] implemented in the Brain Connectivity Toolbox [2]. In this procedure, each connection was swapped between nodes once (on average), while maintaining the network's original degree distribution and ensuring it remained connected.

One disadvantage of topologically randomized networks is the introduction of a disproportionate number of long-range connections, resulting in null networks with markedly increased wiring cost compared to the empirical network. To address this issue, we first computed the empirical wiring cost of a network as the sum of Euclidean distances between its connected nodes [5]. We then generated cost-preserving topologically randomized networks by adding a constraint to the Maslov-Sneppen routine, namely that connection swaps must not alter the original network's cost by more than 1mm. Previous studies report that this leads to surrogate networks that match empirical wiring cost within a 0.1% error margin [6].

Spatially randomized networks are relevant for navigation, which routes information based on local knowledge of network geometry. They are constructed by randomly swapping the spatial positioning of nodes, while maintaining network topology unaltered.

Note 4: Send-receive asymmetries and normality assumptions

Send-receive asymmetry between nodes/subsystems i, j is computed using a one-sample t-test on the distribution $\Delta(i, j, k = 1...K) = C(i, j, k = 1...K) - C(j, i, k = 1...K)$, where C denotes an asymmetric communication matrix and K is the number of subjects. A similar approach is used to compute regional asymmetries based on the node-level distributions $\delta(i, k = 1...K)$ (see *Materials and Methods, Send-receive communication asymmetry measures* for details). However, t-tests mandate an assumption of data normality. We tested the normality of δ for $i = 1...360$ and Δ for $i, j = 1...M$, where $M = 7, 17, 22$ indicate the pairwise asymmetries at different cortical subsystems resolutions. Normality was assessed using a one-sample Kolmogorov-Smirnov test and we considered asymmetries computed for $N = 360$ at 15% connection density. At the regional level, the null hypothesis of normality for 87%, 47% and 85% of the δ distributions could not be rejected at the 5% significance level, for navigation, diffusion and search information, respectively. When accounting for the 360 multiple comparisons using Bonferroni correction, the respective percentages increased to 100%, 91% and 100%. For pairwise asymmetries between subsystems, the null hypothesis of normality could not be rejected at the 5% significance level for 100% of Δ distributions for $M = 7, 17, 22$.

These results suggest that the majority of regional and pairwise communication asymmetries are normally distributed. However, to address possible deviations from normality, we recomputed send-receive asymmetries using a one-sample Wilcoxon rank-sum tests instead of the t-tests. Importantly, the Wilcoxon test is non-parametric and does not require variables to be normally distributed. The resulting w-statistic can be interpreted as an assessment of whether the median of a distribution is significantly smaller or greater than 0. We found that t- and w-statistic based send-receive asymmetries were highly correlated, at both regional and subsystem pairwise levels and for all connection density thresholds. Regional send-receive asymmetries (for $N = 360$) under navigation: Pearson correlation coefficient $r = 0.96, 0.97, 0.97$ for 10%, 15% and 20% connection density thresholds, respectively; under diffusion: $r = 0.68$ for all connection densities; and under search information: $r = 0.89$ for all connection densities. Pairwise subsystem send-receive asymmetries (for $N = 360$ and 15% connection density) under navigation: $r = 0.96, 0.95$ for $M = 17, 22$, respectively; under diffusion: $r = 0.87, 0.89$ for $M = 17, 22$, respectively; and under search information: $r = 0.87, 0.88$ for $M = 17, 22$, respectively. Accordingly, we found that t- and w-statistic send-receive communication asymmetries were consistently correlated with the directionality of effective connectivity (Supplementary Fig. 10a,b).

Note 5: Send-receive asymmetries for connectomes mapped with probabilistic tractography

We sought to determine whether the send-receive asymmetry of the human connectome is robust to alternative connectome reconstruction techniques. To this end, we recomputed the connectomes of the same $K = 200$ subjects using a probabilistic tractography pipeline. Using MRtrix3 [7], we applied multi-shell, multi-tissue constrained spherical deconvolution to estimate the fibre orientation distribution of each white matter voxel [8]. Probabilistic tractography was carried out with the following parameters: iFOD2 algorithm [9], 5×10^6 streamlines, 0.5 mm tracking step-size, 400 mm maximum streamline length and 0.1 fractional anisotropy cutoff for streamline termination. The resulting tractograms were used to construct human connectomes comprising $N = 256, 360, 512$ cortical regions. The obtained connectivity matrices were thresholded at 10%, 15% and 20%

connection density and analysed as weighted networks. Regional and subsystem level send-receive communication asymmetries were computed as for the deterministic connectomes (*Materials and Methods, Send-receive communication asymmetry measures*).

We found that deterministic and probabilistic send-receive asymmetries were strongly correlated for all communication measures, connection density thresholds, parcellation resolutions and cortical subsystem partitions (Supplementary Table II and Supplementary Fig. 11). For instance, deterministic and probabilistic regional asymmetries showed a Pearson correlation coefficient of $r = 0.75, 0.74, 0.77$ under navigation, diffusion and search information, respectively ($N = 360$ at 15% connection density). Similarly, pairwise asymmetries between the two tractography approaches for $M = 22$ subsystems showed $r = 0.70, 0.76, 0.76$ under navigation, diffusion and search information, respectively ($N = 360$ at 15% connection density). All $P < 10^{-30}$.

Communication asymmetries derived from probabilistic connectomes were also correlated with effective connectivity directionality (Supplementary Fig. 10c,d; e.g., $r = 0.64, 0.48, 0.45$ for $M = 17$ under navigation, diffusion and search information, respectively). Interestingly, in most cases, probabilistic asymmetries yielded stronger correlations to effective connectivity directionality than the original deterministic ones. In addition, probabilistic asymmetries did not lead to the drop in association strength for $M = 17$ in fMRI session 2 present in deterministic-based results. However, regional probabilistic-based asymmetries were not associated to the cortical gradient of functional heterogeneity ($P > 0.05$ across all communication measures and parcellation resolutions).

Taken together, these results indicate that send-receive communication asymmetry measures are robust to different tractography pipelines. Regional and pairwise send-receive asymmetries were strongly correlated across deterministic and probabilistic connectome reconstructions and both approaches yielded strong correlations with neural signalling directionality estimated with spectral DCM. Differences in the association strength with effective connectivity directionality and local functional heterogeneity suggest the possibility that deterministic and probabilistic connectomes are better able to capture, respectively, regional and pairwise resting-state functional activity.

Note 6: Senders, receivers and the subcortex

In the main manuscript, we analysed brain networks comprising exclusively of cortico-cortical connections due to challenges in mapping connectomes containing subcortical structures. However, subcortical regions are important mediators of signalling between cortical areas. To investigate the potential impact of subcortical structures to our results, we aimed to reproduce our send-receive asymmetry findings in connectomes including the subcortex. To this end, we mapped the connectomes of the same $K = 200$ subjects using a gray matter parcellation comprising 360 cortical and 14 subcortical regions (left and right thalamus, caudate, putamen, pallidum, hippocampus, amygdala and accumbens-area). The 360 cortical areas were defined according to the HCP MMP1.0 atlas [10], while subcortical structures were derived from the Freesurfer parcellation provided by the HCP (*aparc+aseg* file).

An important challenge in mapping whole-brain connectomes including the subcortex is that certain subcortical structures, in particular the thalamus, are composed of both gray and white matter, functioning both as signal relays and information processing units [11]. However, current parcellations of the subcortex do not provide sufficient detail to differentiate between gray matter nuclei and white matter fibers within subcortical structures. In order to address this issue, we employed a data-driven tractography pipeline with respect to subcortical structures. Subcortical regions were included in both white and gray matter masks. During tractography, subcortical voxels with sub-threshold fractional anisotropy (FA) were interpreted as gray matter, while supra-threshold voxels were considered white matter. Hence, if a streamline tracking through a subcortical region encounters a voxel with low FA (< 0.1), this is interpreted as a portion of gray matter within the subcortical structure, and the streamline's endpoint is assigned to the current subcortical region. However, if a streamline enters and exits a subcortical structure without passing through low FA voxels, this is interpreted as a white matter tract connecting two other (cortical or subcortical) regions. In this case, the streamline endpoint is not assigned to the current subcortical region, providing a data-driven model of subcortical structures as signal relays.

Using this approach, we applied the deterministic tractography algorithm described in *Materials and Methods, Human Connectomes* to map connectivity matrices comprising $N = 360+14 = 374$ nodes. Previously, in order to account for biases towards higher connectivity strength in larger regions, streamline counts between pair of regions were normalized by the product of their surface areas. In this case, since subcortical regions were defined only in volume space, we instead used the pairwise product of regional volumes as a normalization factor. Cortical surface-based parcellations were registered to subject-specific T1-weighted images and the number of voxels comprising gray matter regions was used as an approximation of their volume. This resulted in $K = 200$ subject-level 374×374 weighted connectivity matrices, which were thresholded at 10%, 15% and 20% connection density and analysed as weighted networks. It is noteworthy that subcortical regions ranked amongst the highest-degree nodes in the obtained connectomes (for the average degree across subjects, the thalamus, putamen, hippocampus, pallidum, amygdala, accumbens-area and caudate, ranked, respectively, amongst the top 0.83%,

1.9%, 3.6%, 4.4%, 6.7%, 8.6% and 11.4% most connected nodes).

We began by investigating whether cortical send-receive asymmetries computed on cortico-cortical connectomes were comparable to those derived from connectomes including the subcortex. At the regional level, we found that send-receive asymmetries of cortical regions were consistent across the two sets of connectomes (Supplementary Fig. 12). Pearson correlation coefficients between cortical send-receive asymmetries for connectomes with and without the subcortex: $r = 0.86, 0.84, 0.85$ under navigation; $r = 0.92, 0.93, 0.93$ under diffusion; and $r = 0.90, 0.90, 0.90$ under search information, for 10%, 15% and 20% connection density, respectively (all $P < 10^{-96}$; see Supplementary Fig. 12a,e,i for scatter plots of these associations at 15% connection density). Accordingly, the classification of cortical regions into senders, neutral and receivers was robust to the inclusion of the subcortex: three-way classification accuracy of 69%, 74% and 72% for asymmetries under navigation, diffusion and search information, respectively, at 15% connection density. After the inclusion of subcortical regions, primary sensory regions remained senders (with the exception of V1 for navigation and the right hemisphere M1 for search information), while portions of the precuneus, frontal and prefrontal cortices remained as receivers (Supplementary Fig. 12b,f,j).

Despite these consistencies, the inclusion of subcortical structures did influence the send-receive asymmetry of cortical regions. Supplementary Fig. 12c,g,k shows the difference in regional asymmetries for connectomes with and without the subcortex. Positive differences (shown in red) indicate shifts towards outgoing communication efficiency (increase in send-receive asymmetry), while negative differences (shown in blue) indicate shifts towards incoming communication efficiency (decrease in send-receive asymmetry). In other words, following the inclusion of the subcortex, regions shown in red and blue became relatively more biased towards sending and receiving information, respectively. For navigation (Supplementary Fig. 12c), considering subcortical communication pathways reduced the propensity of visual, somatosensory and auditory cortices towards outgoing communication efficiency. Accordingly, portions of the temporal-parietal-occipital junction, MT+ complex, and medial and dorsolateral cortices had their propensity towards incoming communication efficiency attenuated. Similar patterns of asymmetry differences were observed under diffusion and search information (Supplementary Fig. 12g,k), with the marked distinction that early visual areas (V1 and V2) increased their propensity towards outgoing communication. Importantly, as described above, the majority of cortical regions were consistently classified as senders, neutral or receivers, regardless of whether subcortical structures were present. Hence, these results indicate that subcortical nodes, by mediating communication between cortical areas, influence the intensity (rather than the sign) of cortical send-receive communication asymmetries.

Next, we turned our attention to the send-receive asymmetry of individual subcortical regions. Across all communication measures, all subcortical structures showed a marked bias towards outgoing communication efficiency (Supplementary Fig. 12d,h,l). This is an interesting result, particularly because it opposes the propensity of high-degree regions towards incoming communication reported for diffusion and search information (see *Results, Senders and receivers of the human connectome*). The consistent classification of subcortical regions as senders (with exception of the accumbens-area) potentially reflects their role as inputs of sensory signals to primary cortices.

We also sought to investigate pairwise asymmetries between the subcortex and cortical subsystems. To this end, subcortical regions were grouped into a single subsystem. In keeping with regional results, pairwise asymmetry matrices between cortical subsystems were robust to the inclusion of the subcortex (Supplementary Fig. 13; navigation: $r = 0.89, 0.88$, diffusion: $r = 0.69, 0.76$, search information: $r = 0.71, 0.80$, for $M = 7, 22$, respectively. All $P < 10^{-3}$). Accordingly, individual cortical subsystems showed similar classification as senders and receivers following the addition of subcortical regions (Supplementary Fig. 13b,e,h,l,o,r). Interestingly, a comparison of the send-receive asymmetry of cortical subsystems with (colored horizontal bars) and without the subcortex (colored circles) corroborates the notion that subcortical pathways generally attenuate the communication asymmetry between cortical systems, but do not interfere with their directionality. Once more in agreement with regional results, the subcortex subsystem was consistently classified as a prominent sender.

Finally, we found that send-receive asymmetries between cortical subsystems remained correlated to effective connectivity following the inclusion of the subcortex (Supplementary Fig. 10e,f). Interestingly, associations for $M = 17$ subsystems (Yeo resting-state functional modules [12]) were weakened, while associations for $M = 22$ (HCP contiguous modules [10]) were strengthened, with search information asymmetry leading to correlations as high as $r = 0.68$ (resting-state session 2, 15% connection density).

Note 7: Senders and receivers of non-human connectomes

In this section, we explore send-receive asymmetries computed on symmetrized (undirected) non-human connectomes (*Materials and methods, Symmetrized non-human connectomes*). For sake of conciseness, we refer to those as simply send-receive asymmetries.

We begin by noting that for all non-human connectomes considered, send-receive asymmetries under navigation were not correlated to send-receive asymmetries under diffusion or search information (all $P > 0.05$). As

with the human results, diffusion and search information asymmetries were strongly correlated across species ($r = 0.96, 0.75, 0.75$ for fly, mouse and macaque regional send-receive asymmetries, respectively). Hence, the obtained classification of regions as senders and receivers was different between navigation and random walk based measures. A possible explanation for this disparity lies in the high connection density of the non-human connectomes (fly: 83%, 89%, mouse: 53%, 70%, macaque: 66%, 79%, directed (original) and undirected (symmetrized) connection densities, respectively). In undirected networks (as obtained after connectome symmetrization), there is no navigation asymmetry between directly connected node pairs (if $W(i,j) = W(j,i) \neq 0$ it follows that $E_{nav}(i,j) = E_{nav}(j,i)$. See *Materials and Methods, Navigation efficiency*). This may lead to a decrease in the biological relevance of navigation asymmetries in connectomes for which most regions are directly connected. Meanwhile, the stochastic nature of communication measures based on random walks gives rise to send-receive asymmetries even in densely connected networks. In support of this notion, we found that senders and receivers identified under diffusion and search information were consistent with the putative functional roles of different brain regions in the fly, mouse and macaque. Moreover, send-receive asymmetries computed on binary connectomes yield the most biologically relevant classification of senders and receivers. Given the agreement between diffusion and search information, we focus on describing the results obtained for binary diffusion send-receive asymmetry in the following paragraphs.

A limitation of non-human connectome analyses is the lack of subject-level data. Importantly, this shortcoming precludes the use of the same send-receive asymmetry framework developed for humans. As a result, send-receive asymmetry for these species are not defined statistically at the level of individual regions or pairs of regions. To address this limitation, we partitioned the nodes comprising non-human connectomes into M previously defined subsystems of the fly, mouse and macaque nervous systems. We downsampled the node-level $N \times N$ send-receive asymmetry matrices into subsystem-level $M \times M$ matrices. As a result, each subsystem pair is associated to a distribution of node-level pairwise asymmetries. For each subsystem pair i, j, we computed whether the mean of their distribution of node-level pairwise asymmetries was significantly larger than 0 by means of a one-sample t-test. Send-receive asymmetry between subsystems was defined as the resulting t-statistic. Note that while human asymmetries were statistically defined based on cross-subject distributions, here we define non-human subsystem asymmetries across node pairs. This allowed us to statistically test hypotheses on the agreement between communication asymmetry and the putative functional roles of subsystems of non-human connectomes.

Supplementary Fig. 14a shows the regional send-receive diffusion asymmetry of the 49 neuronal populations comprising the fly connectome. Following previous work on decentralized communication in the fly connectome, we classified neuronal populations into three groups: (i) sensors (ii) effectors and (iii) others [13]. Sensor nodes are involved in the processing and transducing of sensory information, and are thus hypothesised to be senders. Effectors are conjectured to integrate signals from different neuronal populations in order to coordinate motor execution, and are thus hypothesised to be receivers. In line with these hypotheses, 4 out of 6 sensor nodes were classified as senders (positive send-receive asymmetry) while all effector nodes were classified as receivers (negative send-receive asymmetry). Additionally, we found that the mean sensor $\rightarrow$ effector and other $\rightarrow$ effector asymmetries were significantly larger than 0 ($P = 1 \times 10^{-9}, 5 \times 10^{-24}$, respectively. Supplementary Fig. 14b). The average sensor $\rightarrow$ other asymmetry was positive, but not significantly larger than 0 ($P = 0.11$). Asymmetries between sensor $\rightarrow$ effector and other $\rightarrow$ effector nodes were not statistically different (two-sample t-test $P = 0.46$). Together, these findings indicate that diffusion send-receive asymmetry recapitulates the putative functional roles of neural populations in the fly connectome by characterizing sensor nodes as senders and effector nodes as receivers (Supplementary Fig. 14c).

Supplementary Fig. 14d shows the regional send-receive diffusion asymmetry of regions comprising the mouse connectome (values were averaged across homotopic regions). Consistent with the human results, we found that primary auditory (AUDp) and visual (VISp) areas were senders (positive send-receive asymmetry). However, primary motor (MOs) and sensory (SSp) were classified as receivers. We partitioned brain regions into 8 modules according to a previously established modular decomposition of the mouse connectome [14]. The 8 modules are: somatosensory-motor (SS-M), brainstem-cerebellum (BS-CE), auditory (AU), visual (VI), olfactory (OL), hippocampal (HI), hypothalamic (HY), and high-participation (Hi-Par, i.e., regions characterized by a large proportion of intermodule connections that could not be consistently assigned to a single module). Generally, subsystem associated with the processing of sensory information were senders (olfactory, visual and auditory), with the exception of somatosensory-motor (Supplementary Fig. 14e,f). The brainstem-cerebellum subsystem was the most prominent sender, potentially reflecting the role of subcortical structures in relaying sensory signals to primary cortices. In agreement with the human findings, regions with diverse intermodule connectivity (high participation) were prominent receivers. Together, these results indicate that the undirected topology of the mouse connectome leads to biases in outgoing information for auditory, visual and olfactory regions. Meanwhile, sensory and motor areas may be more dependent on axonal directionality to propagate information to higher-order areas of the mouse cortex.

Finally, Supplementary Fig. 14g shows the regional send-receive diffusion asymmetry of the 29 regions comprised in the macaque connectome. In agreement with

results reported for the human connectome, areas of the sensory (area 2), visual (V1 and V2) and motor (F1 and ProM) cortices were classified as senders (positive send-receive asymmetry), while portions of the frontal (8m and 8l) and prefrontal cortices (46d, 9/46v, 9/46d) were classified as receivers (negative send-receive asymmetry). Supplementary Fig. 15 compares the cortical projections of human and macaque send-receive asymmetries and provides further evidence for consistencies between the results of the two species: expanses of the occipital cortex and sensory-motor strip are classified as senders, while portions of the prefrontal cortex are classified as receivers. Certain differences between the two species were observed: higher-order visual areas such as V4 were receivers in humans but senders in the macaque, while expanses of the temporal lobe appear as senders in humans and receivers in the macaque. Cortical regions were assigned to 6 modules according to a previously established partition of the macaque connectome [15]. Occipital and prefrontal modules were the most prominent senders and receivers, respectively (Supplementary Fig. 14h,i). Taken together, these findings indicate that the interaction between decentralized network communication measures and connectome topology leads to send-receive asymmetries that recapitulate uni- to heteromodal cortical hierarchies in both human and macaque connectomes. These results suggest that topological properties of undirected connectomes contributing to neural signalling directionality are phylogenetically conserved across higher primates.

It is important to notice that these analyses constitute a first account of send-receive asymmetry in non-human connectomes. Further work is necessary to consolidate the results presented here. Potential future steps in this direction include (i) a conceptualization of statistically defined send-receive asymmetry inferred from single-subject networks and (ii) a better understanding motivating the observed biological relevance of binary diffusion-based communication asymmetries.

---

[1] V. D. Blondel, J.-L. Guillaume, R. Lambiotte, and E. Lefebvre, “Fast unfolding of communities in large networks,” *Journal of statistical mechanics: theory and experiment*, 2008.

[2] M. Rubinov and O. Sporns, “Complex network measures of brain connectivity: uses and interpretations,” *Neuroimage*, vol. 52, pp. 1059–69, Sep 2010.

[3] R. Guimerà and L. A. Nunes Amaral, “Functional cartography of complex metabolic networks,” *Nature*, vol. 433, pp. 895–900, Feb 2005.

[4] S. Maslov and K. Sneppen, “Specificity and stability in topology of protein networks,” *Science*, vol. 296, pp. 910–3, May 2002.

[5] R. F. Betzel, A. Avena-Koenigsberger, J. Goñi, Y. He, M. A. de Reus, A. Griffa, P. E. Vértes, B. Mišic, J.-P. Thiran, P. Hagmann, M. van den Heuvel, X.-N. Zuo, E. T. Bullmore, and O. Sporns, “Generative models of the human connectome,” *Neuroimage*, vol. 124, pp. 1054–64, Jan 2016.

[6] C. Seguin, M. P. van den Heuvel, and A. Zalesky, “Navigation of brain networks,” *Proc Natl Acad Sci U S A*, vol. 115, pp. 6297–6302, 06 2018.

[7] J.-D. Tournier, F. Calamante, and A. Connelly, “Mrtrix: Diffusion tractography in crossing fiber regions,” *International Journal of Imaging Systems and Technology*, vol. 22, 03 2012.

[8] B. Jeurissen, J.-D. Tournier, T. Dhollander, A. Connelly, and J. Sijbers, “Multi-tissue constrained spherical deconvolution for improved analysis of multi-shell diffusion mri data,” *Neuroimage*, vol. 103, pp. 411–26, Dec 2014.

[9] J. D. Tournier, F. Calamante, and A. Connelly, “Improved probabilistic streamlines tractography by 2nd order integration over fibre orientation distributions,” in *Proceedings of the international society for magnetic resonance in medicine*, vol. 18, p. 1670, 2010.

[10] M. F. Glasser, T. S. Coalson, E. C. Robinson, C. D. Hacker, J. Harwell, E. Yacoub, K. Ugurbil, J. Andersson, C. F. Beckmann, M. Jenkinson, S. M. Smith, and D. C. Van Essen, “A multi-modal parcellation of human cerebral cortex,” *Nature*, vol. 536, pp. 171–178, 08 2016.

[11] R. W. Guillery and S. M. Sherman, “Thalamic relay functions and their role in corticocortical communication: generalizations from the visual system,” *Neuron*, vol. 33, pp. 163–75, Jan 2002.

[12] B. T. T. Yeo, F. M. Krienen, J. Sepulcre, M. R. Sabuncu, D. Lashkari, M. Hollinshead, J. L. Roffman, J. W. Smoller, L. Zöllei, J. R. Polimeni, B. Fischl, H. Liu, and R. L. Buckner, “The organization of the human cerebral cortex estimated by intrinsic functional connectivity,” *J Neurophysiol*, vol. 106, pp. 1125–65, Sep 2011.

[13] J. C. Worrell, J. Rumschlag, R. F. Betzel, O. Sporns, and B. Mišić, “Optimized connectome architecture for sensory-motor integration,” *Netw Neurosci*, vol. 1, no. 4, pp. 415–430, 2018.

[14] M. Rubinov, R. J. F. Ypma, C. Watson, and E. T. Bullmore, “Wiring cost and topological participation of the mouse brain connectome,” *Proc Natl Acad Sci U S A*, vol. 112, pp. 10032–7, Aug 2015.

[15] N. T. Markov, M. M. Ercsey-Ravasz, A. R. Ribeiro Gomes, C. Lamy, L. Magrou, J. Vezoli, P. Misery, A. Falchier, R. Quilodran, M. A. Gariel, J. Sallet, R. Gamanut, C. Huissoud, S. Clavagnier, P. Giroud, D. Sappey-Marinier, P. Barone, C. Dehay, Z. Toroczkai, K. Knoblauch, D. C. Van Essen, and H. Kennedy, “A weighted and directed interareal connectivity matrix for macaque cerebral cortex,” *Cereb Cortex*, vol. 24, pp. 17–36, Jan 2014.

[16] D. S. Margulies, S. S. Ghosh, A. Goulas, M. Falkiewicz, J. M. Huntenburg, G. Langs, G. Bezgin, S. B. Eickhoff, F. X. Castellanos, M. Petrides, E. Jefferies, and J. Smallwood, “Situating the default-mode network along a principal gradient of macroscale cortical organization,” *Proc Natl Acad Sci U S A*, vol. 113, pp. 12574–12579, 11 2016.

[17] M. Xia, J. Wang, and Y. He, “Brainnet viewer: a network visualization tool for human brain connectomics,” *PLoS One*, vol. 8, no. 7, p. e68910, 2013.

	S	N	R
S	86	15	24
N	35	20	36
R	61	22	61

(a) Navigation (rows) and diffusion (columns): $Acc = 0.46$.

	S	N	R
S	70	15	40
N	27	13	51
R	43	22	79

(b) Navigation and SI: $Acc = 0.44$.

	S	N	R
S	140	37	5
N	0	13	44
R	0	0	121

(c) Diffusion and SI: $Acc = 0.76$.

	S	$\neg S$
S	129	101
$\neg S$	49	81

(d) Nav & diff: $Acc = 0.58$, $P = 0.001$.

	R	$\neg R$
R	157	56
$\neg R$	82	65

(e) Nav & diff: $Acc = 0.67$, $P = 6 \times 10^{-4}$.

	S	$\neg S$
S	155	75
$\neg S$	65	65

(f) Nav & SI: $Acc = 0.61$, $P = 0.002$.

	R	$\neg R$
R	124	89
$\neg R$	66	81

(g) Nav & SI: $Acc = 0.57$, $P = 0.014$.

	S	$\neg S$
S	178	0
$\neg S$	42	140

(h) Diff & SI: $Acc = 0.88$, $P = 3 \times 10^{-62}$.

	R	$\neg R$
R	190	49
$\neg R$	0	121

(i) Diff & SI: $Acc = 0.86$, $P = 5 \times 10^{-56}$.

TABLE I. Comparison of the classification of cortical regions as senders (S), neutral (N) and receivers (R) across network communication measures. The classification accuracy (Acc) is computed as the sum of values in the main diagonal (number of consistently classified regions) divided by the sum of values in the table (total number of regions). **(a-c)** Three-way contingency tables of the classification obtained from the send-receive asymmetries of two communication measures. Measures listed first and second in the captions have their classes displayed in the rows and columns of the tables, respectively. **(d-i)** Two-way contingency tables of the classification obtained from the send-receive asymmetries of two communication measures. In this case, for each pair of measures, regions are classified as sender (S) or not sender ($\neg S$), and receiver (R) or not receiver ($\neg R$). P-values obtained from Fisher's exact test were used to examine the significance of the association between the classifications of two measures.

Parcellation resolution	Navigation			Diffusion			Search Info		
	10% CD	15% CD	20% CD	10%	15%	20%	10%	15%	20%
256	0.75	0.71	0.68	0.71	0.72	0.71	0.74	0.74	0.75
360	0.77	0.75	0.73	0.75	0.74	0.74	0.77	0.77	0.77
512	0.74	0.72	0.70	0.67	0.66	0.65	0.65	0.76	0.76

TABLE II. Pearson correlation coefficients between deterministic and probabilistic send-receive asymmetries for different communication measures, connection density (CD) thresholds and parcellation resolutions. All associated $P < 10^{-30}$.

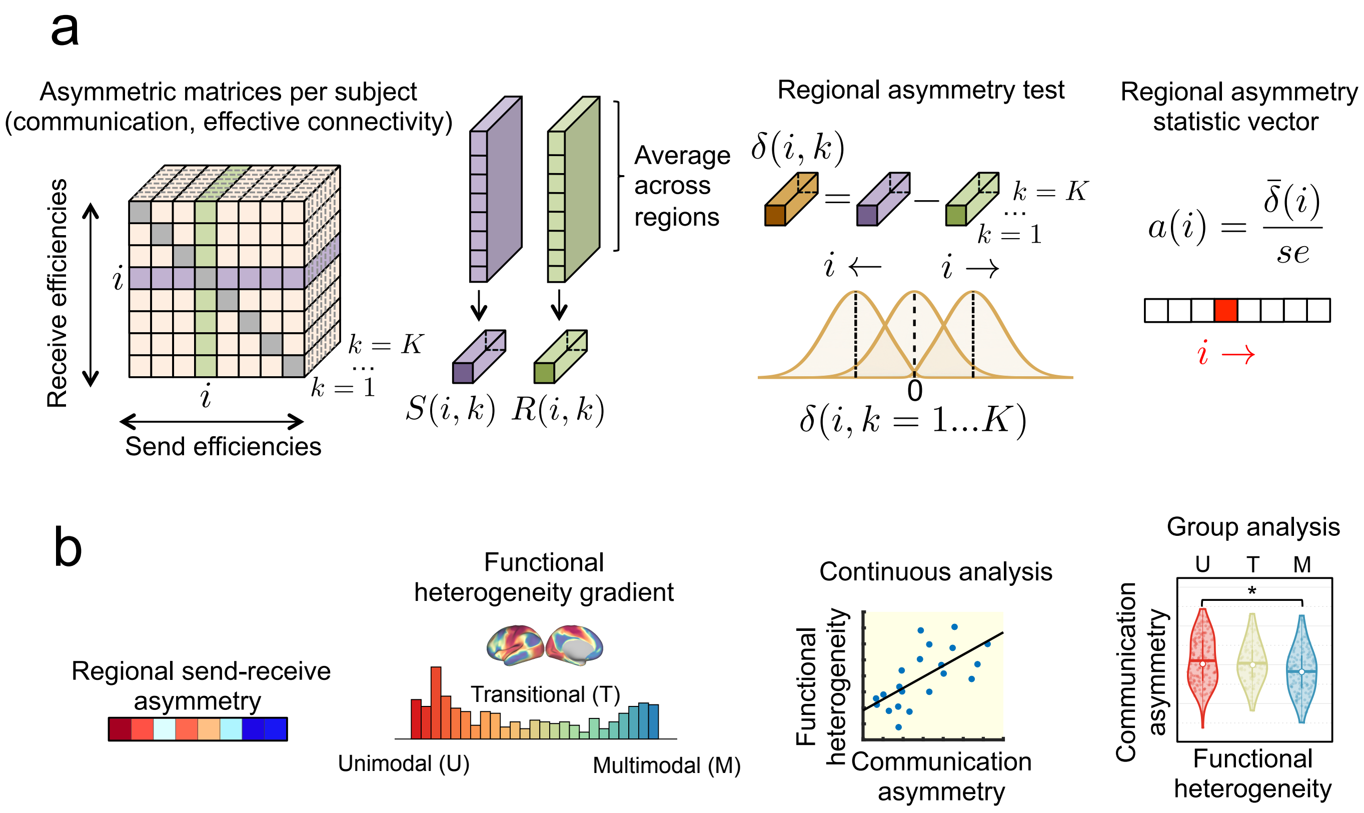

Supplementary Fig. 1. Methodology overview of regional send-receive asymmetry analyses. **(a)** Schematic of the regional communication asymmetry test as described in *Materials and Methods, Send-receive communication asymmetry measures.* **(b)** Schematic of the comparison between regional send-receive asymmetry and the Margulies' gradient of functional heterogeneity [16]. Cortical regions are divided into unimodal, transitional and multimodal based on their placement along the functional gradient, as per described in *Materials and Methods, Cortical gradient of functional heterogeneity.* Comparisons between send-receive asymmetry and functional heterogeneity are performed by means of continuous (i.e., linear correlation) and group-wise (i.e., between group differences) analyses.

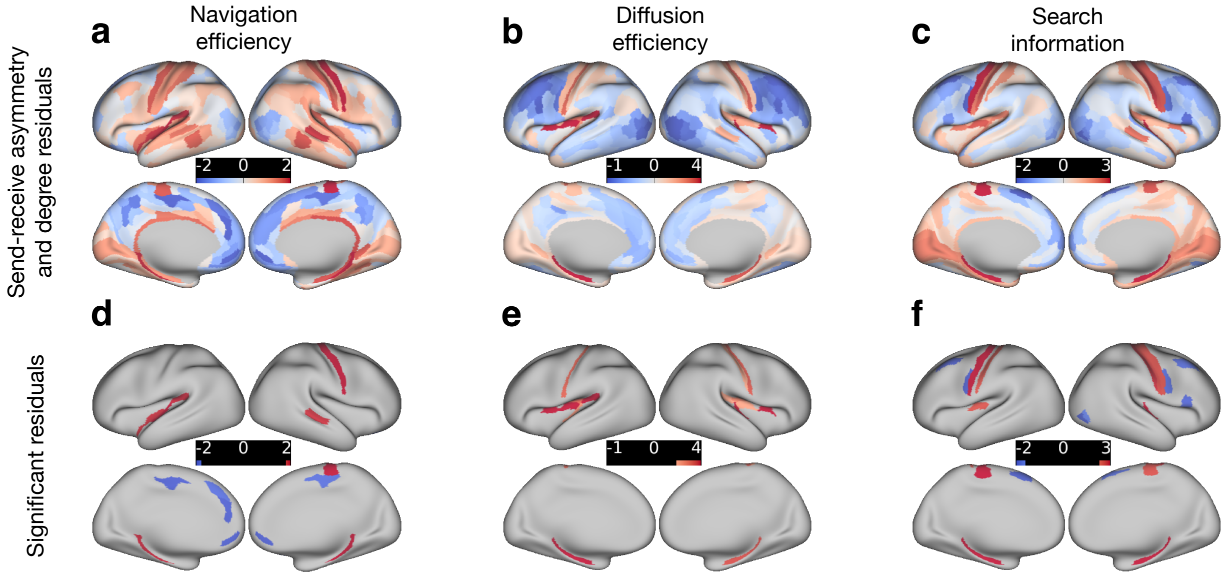

Supplementary Fig. 2. Standardized residuals ($\hat{\epsilon}$) obtained from regressing out node degree from send-receive asymmetry ($N = 360$ at 15% connection density) under **(a,d)** navigation, **(b,e)** diffusion, and **(c,f)** search information. Regions shown in red ($\hat{\epsilon} > 0$) and blue ($\hat{\epsilon} < 0$) are, respectively, stronger senders and receivers than expected based on their degree alone. Regions with statistically significant residuals ($|\hat{\epsilon}| > 1.96$) are highlighted in the bottom row.

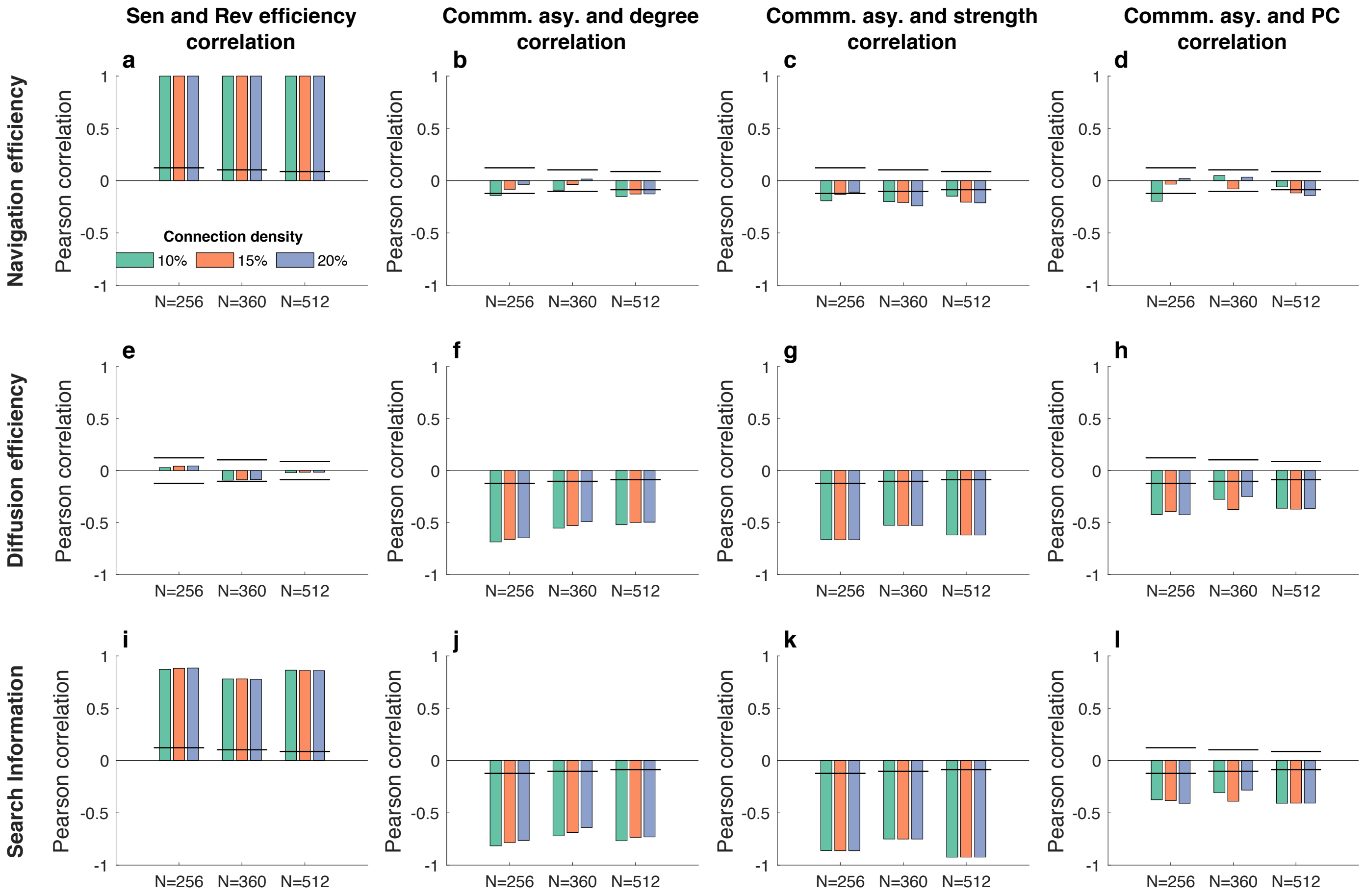

Supplementary Fig. 3. Replication analyses regarding sending efficiency, receiving efficiency and send-receive communication asymmetry for $N = 256, 360, 512$ parcellation resolutions and 10%, 15% and 20% connection density thresholds. Vertical axes indicate the Pearson correlation's r, while black horizontal lines mark the effect size correspondent to a correlation with $P = 0.05$ for each N. **(a)** Correlation between sending and receiving navigation efficiencies. **(b)** Correlation between regional navigation asymmetry and node degree (averaged across the connectomes of all participants). **(c)** Same as b, but for node strength. **(d)** Same as a, but for node participation coefficient.**(e-h)** Same as a-d, but for diffusion efficiency. **(i-l)** Same as a-d, but for search information.

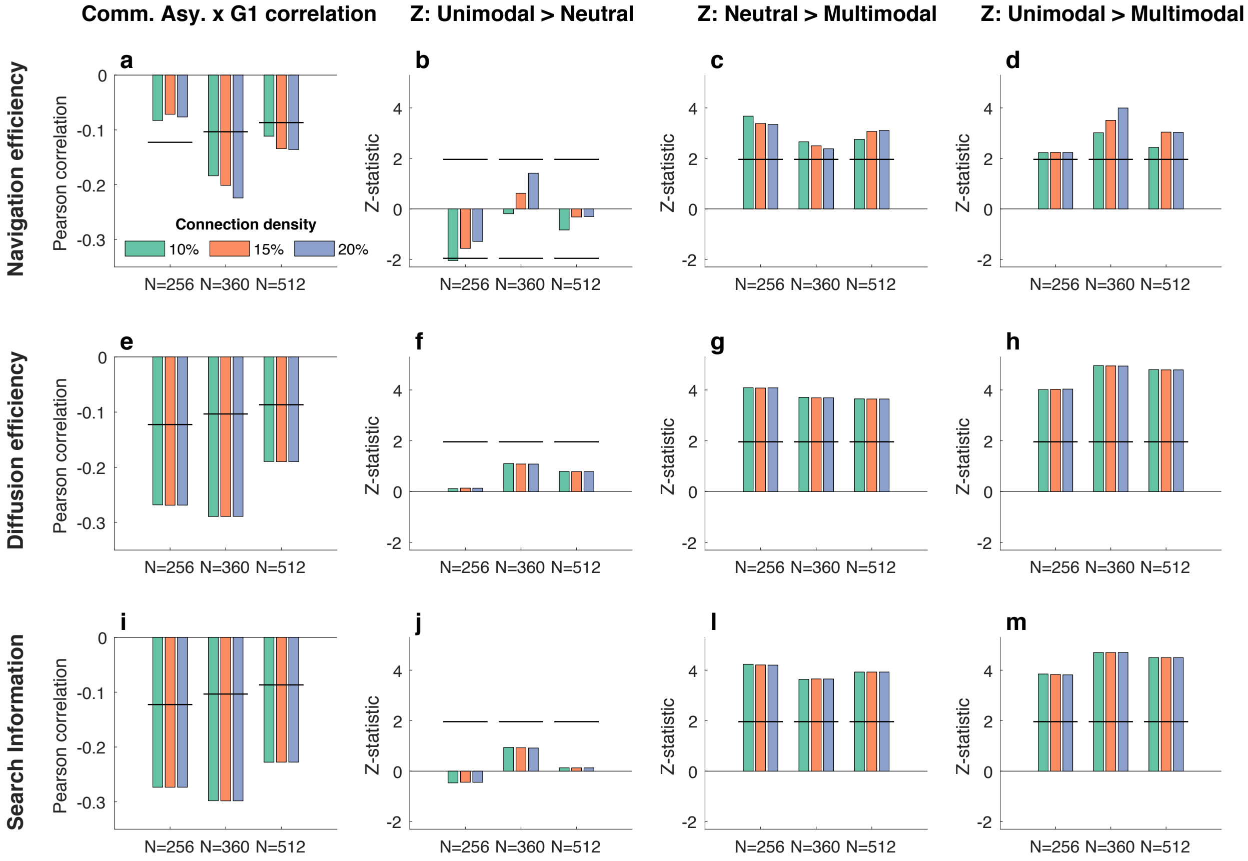

Supplementary Fig. 4. Replication analyses regarding the relationship between communication asymmetry and functional heterogeneity for $N = 256, 360, 512$ parcellation resolutions and 10%, 15% and 20% connection density thresholds. The obtained results are consistent across parcellations and connection densities, with the exception of the lack of correlation between send-receive asymmetry and the gradient of functional heterogeneity for $N = 256$. **(a)** Pearson correlation between regional navigation asymmetry and functional heterogeneity. Black horizontal lines mark the effect size correspondent to a correlation with $P = 0.05$ for each N. **(b)** Z-statistic from a two-sided Wilcoxon test evaluating the hypothesis that the median navigation asymmetry of unimodal regions is larger than that of neutral regions. Black horizontal lines mark the value of a Z-statistic correspondent to $P = 0.05$. **(c)** Same as b, but for neutral and multimodal regions. **(d)** Same as b, but for unimodal and multimodal regions. **(e-h)** Same as a-d, but for diffusion efficiency. **(i-m)** Same as a-d, but for search information.

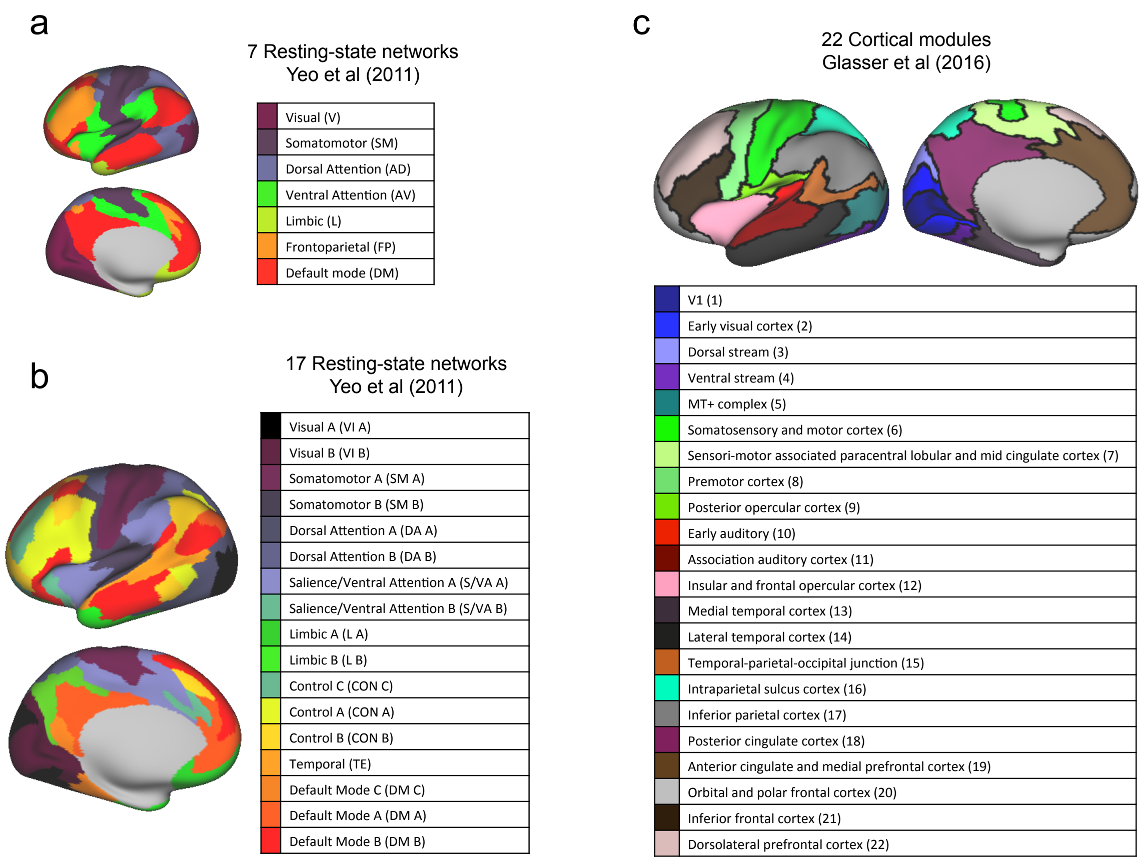

Supplementary Fig. 5. Definition of the $M = 7, 17, 22$ cortical subsystems utilized in sections *Send-receive communication asymmetries of cortical subsystems Send-receive communication asymmetry and effective connectivity.*

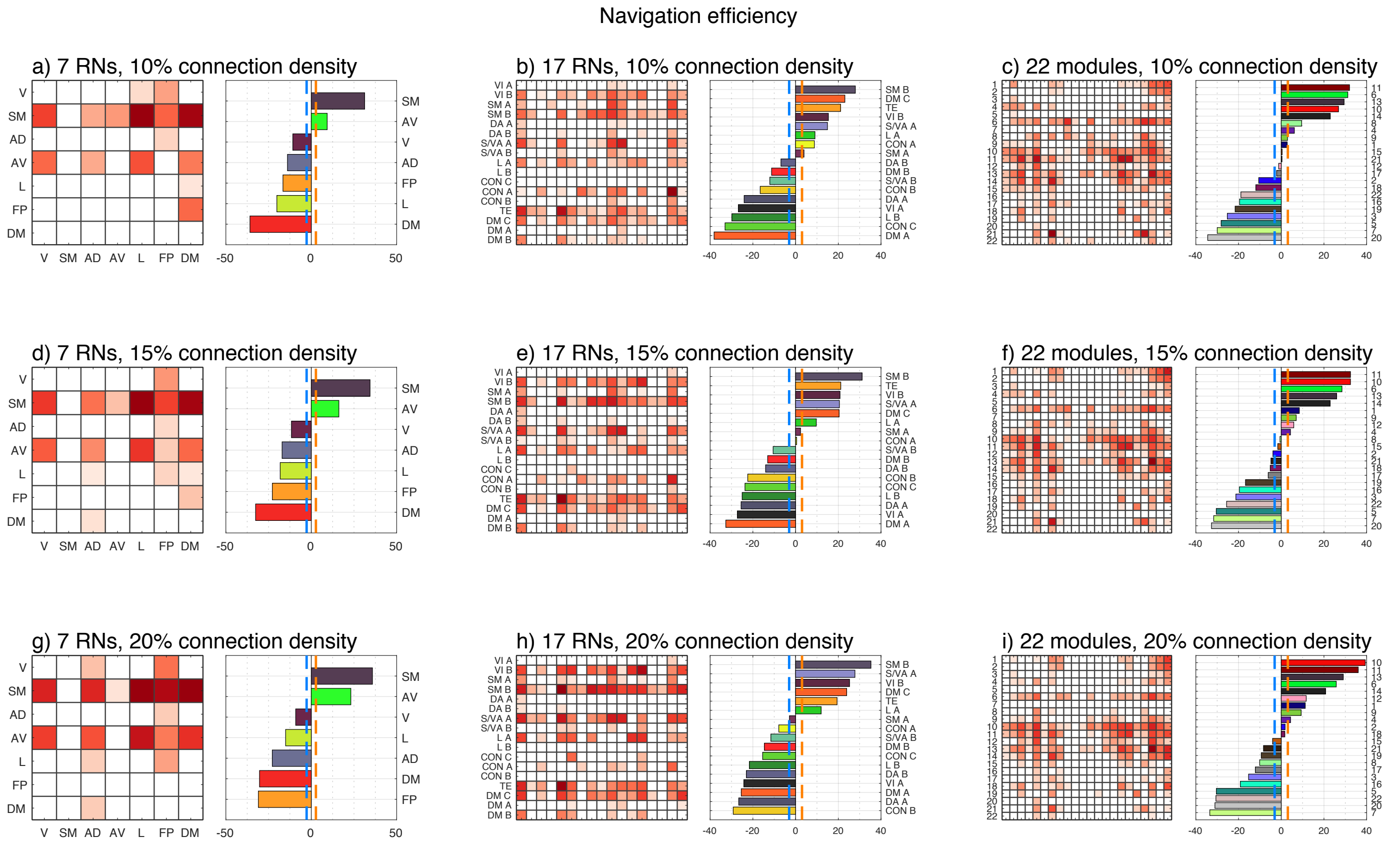

Supplementary Fig. 6. Send-receive navigation asymmetry of cortical subsystems for $M = 7, 17, 22$ and 10%, 15% and 20% connection density thresholds. Send-receive asymmetry matrices were thresholded to display only statistically significant values, while accounting for multiple comparisons. For ease of visualization and without loss of information (since $A(i,j) = -A(j,i)$), negative values were omitted. Thus, $A(i,j) > 0$ denotes that communication takes place more efficiently from i to j than from j to i.

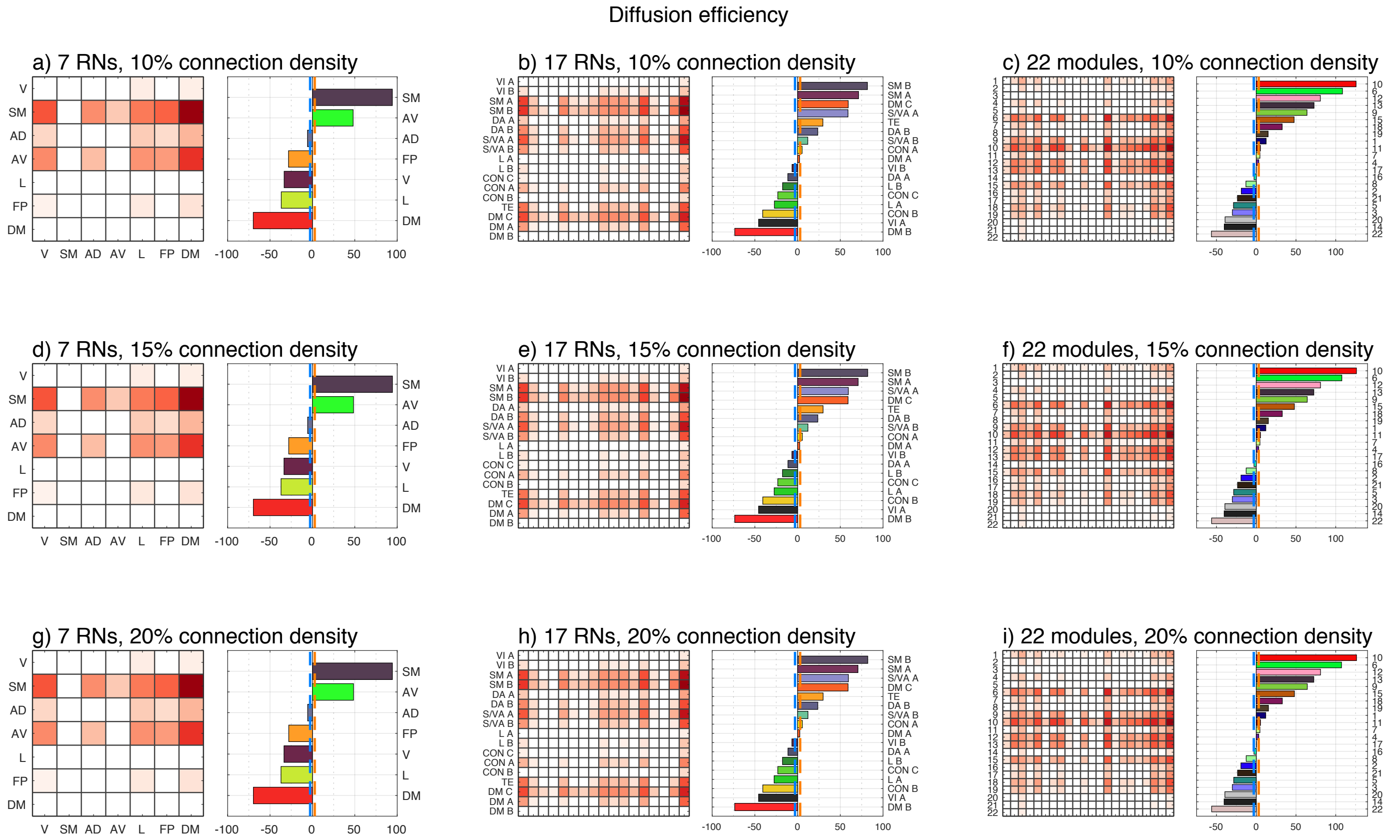

Supplementary Fig. 7. Send-receive diffusion asymmetry of cortical subsystems for $M = 7, 17, 22$ and 10%, 15% and 20% connection density thresholds. Send-receive asymmetry matrices were thresholded to display only statistically significant values, while accounting for multiple comparisons. For ease of visualization and without loss of information (since $A(i,j) = -A(j,i)$), negative values were omitted. Thus, $A(i,j) > 0$ denotes that communication takes place more efficiently from i to j than from j to i.

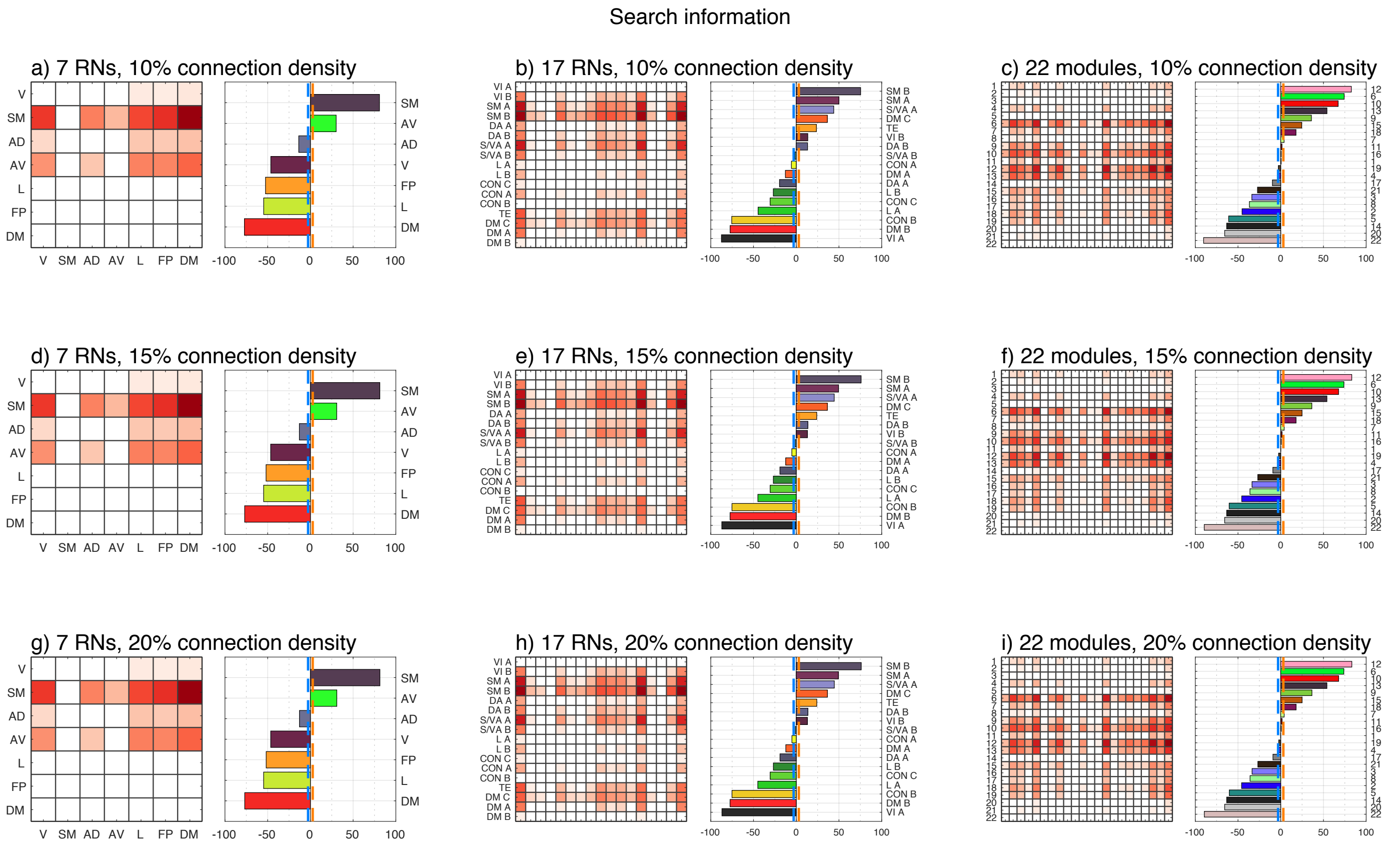

Supplementary Fig. 8. Send-receive search information asymmetry of cortical subsystems for $M = 7, 17, 22$ and 10%, 15% and 20% connection density thresholds. Send-receive asymmetry matrices were thresholded to display only statistically significant values, while accounting for multiple comparisons. For ease of visualization and without loss of information (since $A(i,j) = -A(j,i)$), negative values were omitted. Thus, $A(i,j) > 0$ denotes that communication takes place more efficiently from i to j than from j to i.

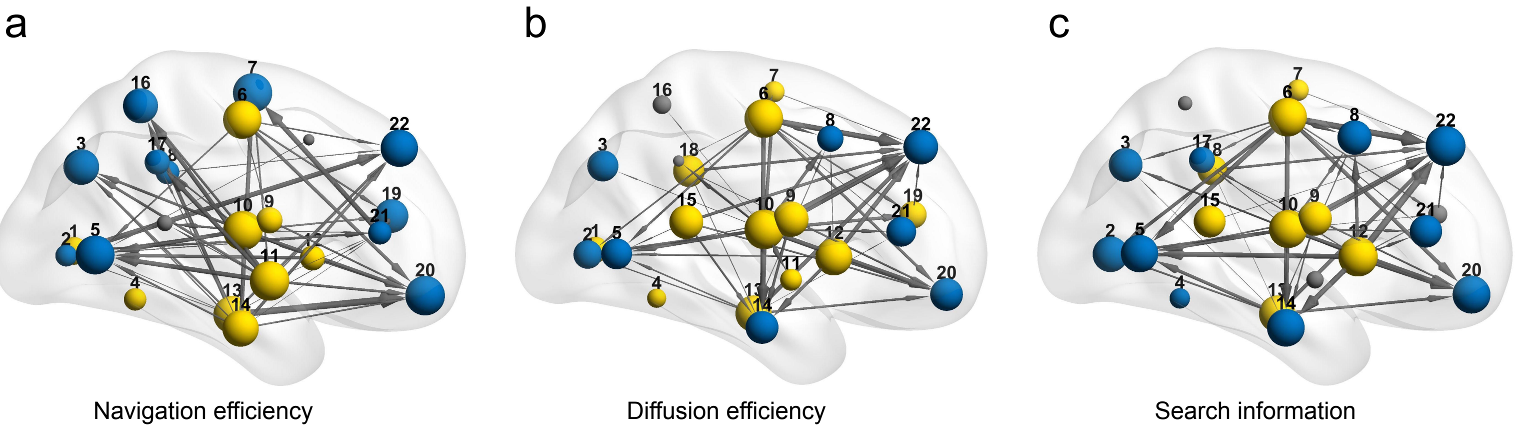

Supplementary Fig. 9. Network visualization of the 10% strongest pairwise send-receive asymmetries between $M = 22$ cortical subsystems ($N = 360$ at 15% structural connection density threshold) under **(a)** navigation, **(b)** diffusion and **(c)** search information. A directed connection between from subsystem i to subsystem j indicates that the communication efficiency from i to j is significantly higher than the communication efficiency from j to i. Connection width is proportional to the strength of the send-receive asymmetry. Subsystems classified as senders, neutral and receivers are shown in yellow, gray and blue, respectively. Visualization developed with BrainNet Viewer [17].

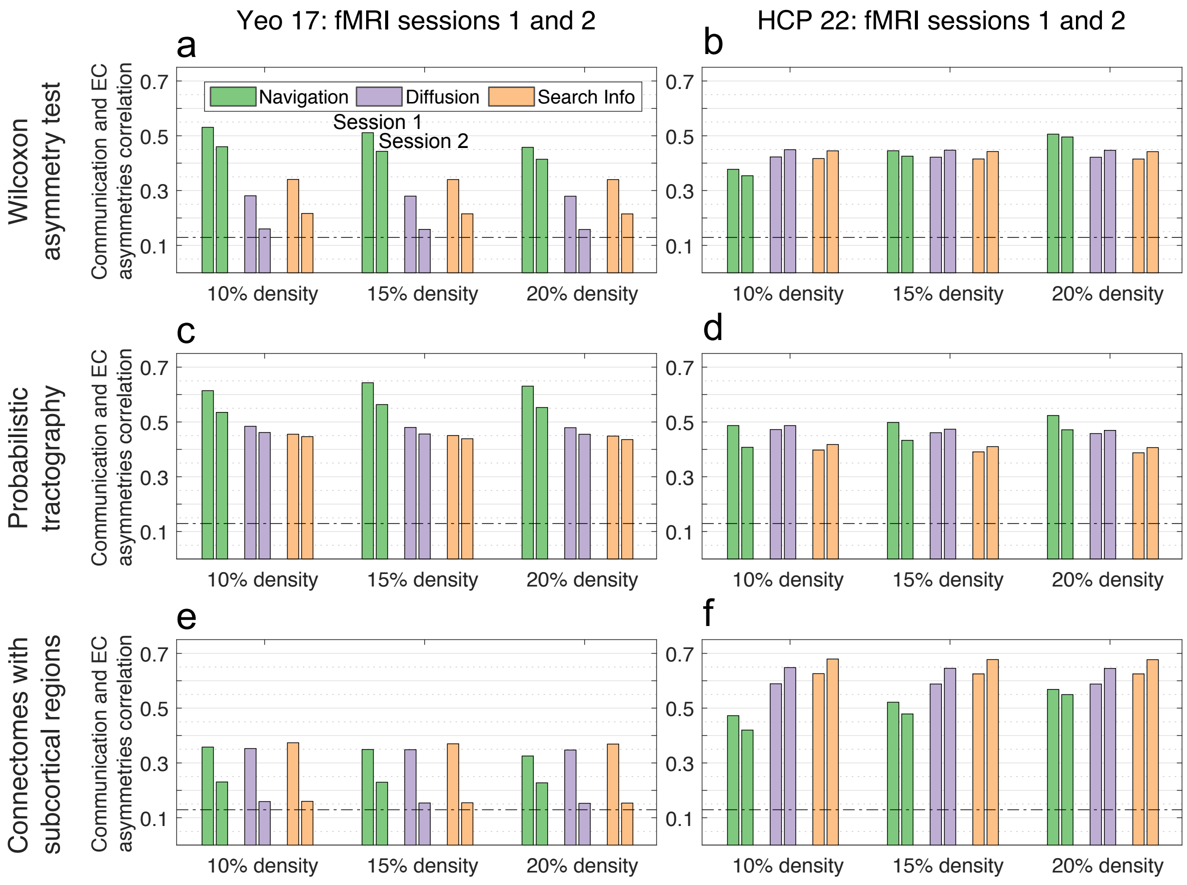

Supplementary Fig. 10. Relationship between send-receive asymmetry and directionality of effective connectivity across a range of methodological settings ($N = 360$). Bars denote the Pearson correlation coefficients between send-receive and effective connectivity asymmetries for $M = 17$ cortical subsystems. Bars are colored according to the three communication measures: i) navigation (green), ii) diffusion (violet), and iii) search information (beige). Correlations were computed for two independent resting-state fMRI sessions (Sessions 1 and 2) and multiple structural connection density thresholds (10, 15 and 20%). Significance threshold of $P < 0.05$ is indicated with a dotted line. **(a,b)** Non-parametric definition of send-receive asymmetry for $M = 17, 22$ cortical subsystems. **(c,d)** Send-receive asymmetry computed on connectomes derived using probabilistic tractography for $M = 17, 22$ cortical subsystems. **(e,f)** Send-receive asymmetry computed on connectomes including subcortical regions for $M = 17, 22$ cortical subsystems.

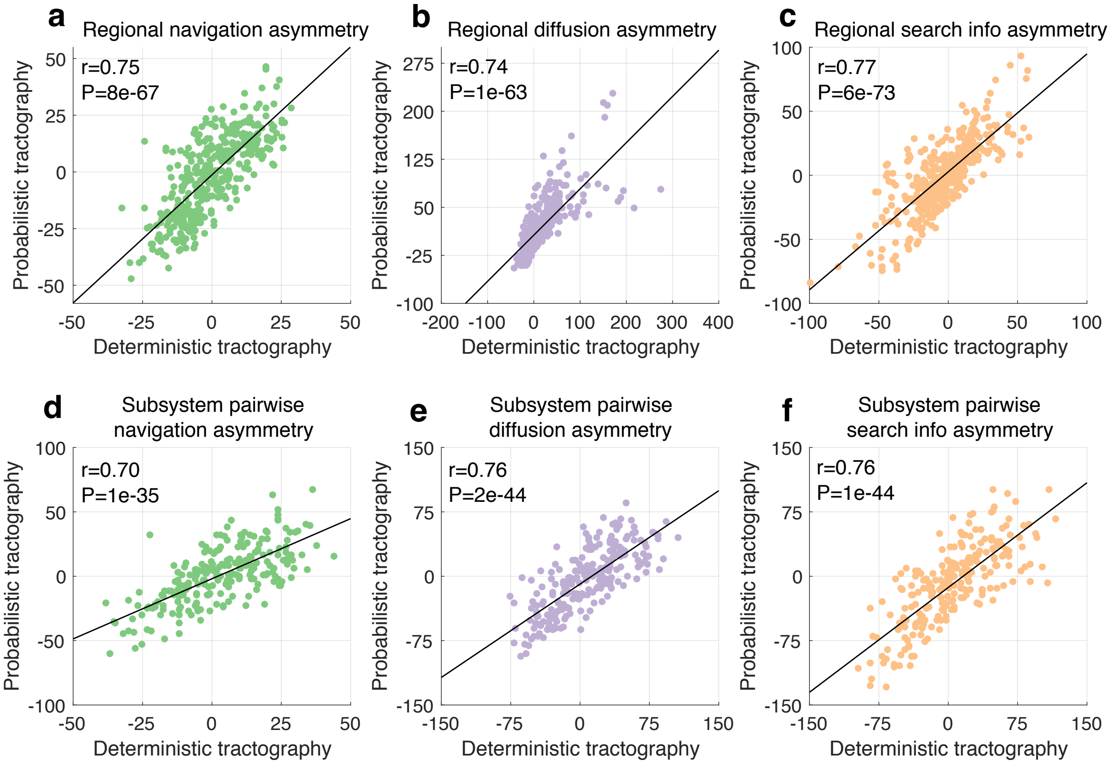

Supplementary Fig. 11. Scatter plots showing the relationship between send-receive asymmetries derived from deterministic and probabilistic connectomes ($N = 360$). **(a,b,c)** Deterministic and probabilistic regional send-receive asymmetries for navigation, diffusion and search information, respectively. **(d,e,f)** Subsystem pairwise send-receive asymmetry ($M = 22$) for navigation, diffusion and search information, respectively. r: Pearson correlation coefficient, P: associated P-value.

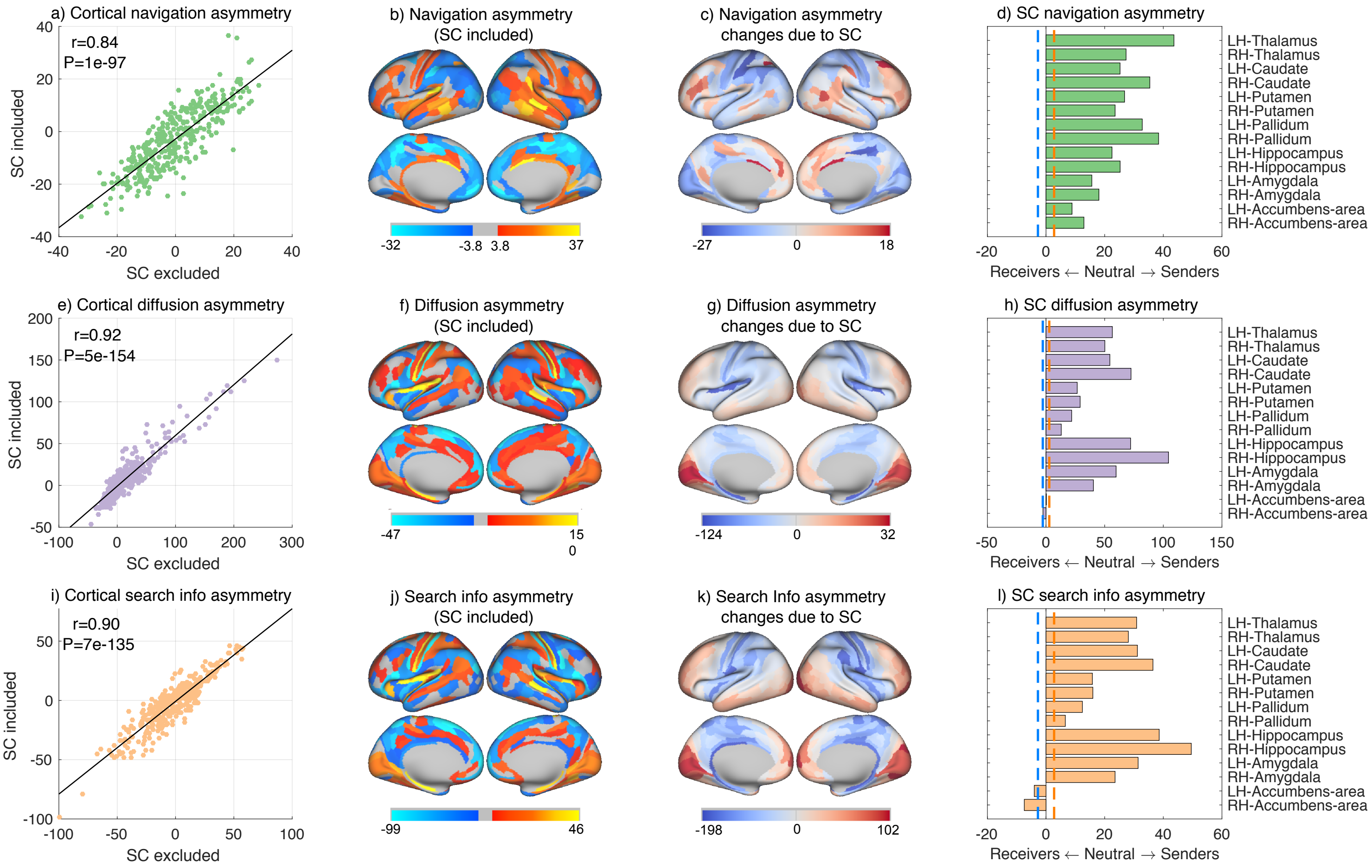

Supplementary Fig. 12. Regional send-receive asymmetry of human connectomes including subcortical regions. **(a)** Scatter plot of cortical navigation asymmetries computed from connectomes excluding subcortical structures (horizontal axis) and including subcortical structures (vertical axis). r: Pearson correlation coefficient, P: associated P-value. **(b)** Cortical navigation asymmetry computed from connectome including subcortical structures. Regions associated with significant send-receive asymmetry are classified as putative senders (orange) and receivers (blue). Regions colored gray are neutral and do not show significant send-receive asymmetry. **(c)** Differences in cortical navigation asymmetry following the inclusion of subcortical structures to the connectome. Positive differences (shown in red) indicate shifts towards outgoing communication efficiency, while negative differences (shown in blue) indicate shifts towards incoming communication efficiency. **(d)** Navigation asymmetry of subcortical regions. Dashed vertical lines indicate a significant bias towards outgoing (orange) and incoming (blue) communication efficiency. **(e–h)** Same as (a–d) for diffusion efficiency. **(i–l)** Same as (a–d) for search information.

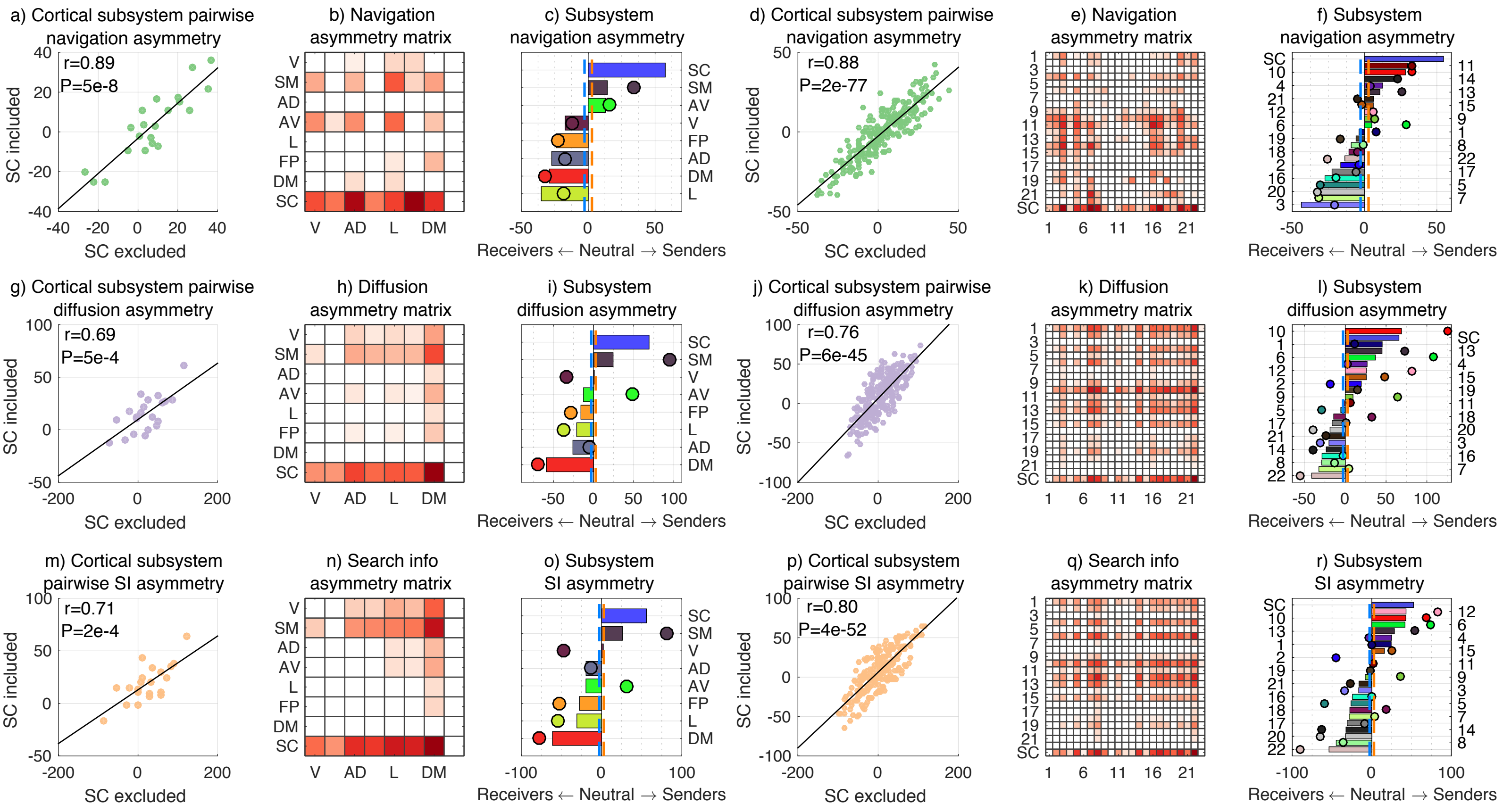

Supplementary Fig. 13. Pairwise subsystem send-receive asymmetry of human connectomes including subcortical regions. **(a)** Scatter plot of pairwise cortical subsystem ($M = 7$) navigation asymmetries computed from connectomes excluding subcortical structures (horizontal axis) and including subcortical structures (vertical axis). r: Pearson correlation coefficient, P: associated P-value. **(b)** Navigation asymmetry matrix from connectomes including the subcortex. Subcortical regions were grouped to form a single subsystem (SC), resulting in $M = 7 + 1$ subsystems. A matrix element $A(i, j) > 0$ denotes that communication occurs more efficiently from i to j than from j to i. Send-receive asymmetry values that did not survive multiple comparison correction were suppressed and appear as white cells. For ease of visualization and without loss of information (since $A(i, j) = -A(j, i)$), negative values were omitted. **(c)** Subsystems ($M = 7 + 1$) ranked by propensity to send (top) or receive (bottom) information under navigation. Send-receive asymmetries computed for connectomes including and excluding the subcortex are shown as bars and circles, respectively. Dashed vertical lines indicate a significant bias towards outgoing (orange) and incoming (blue) communication efficiency. **(d–f)** Same as (a–c) but for $M = 22 + 1$ subsystems. **(g–l)** Same as (a–f) for diffusion. **(g–l)** Same as (m–r) for search information.

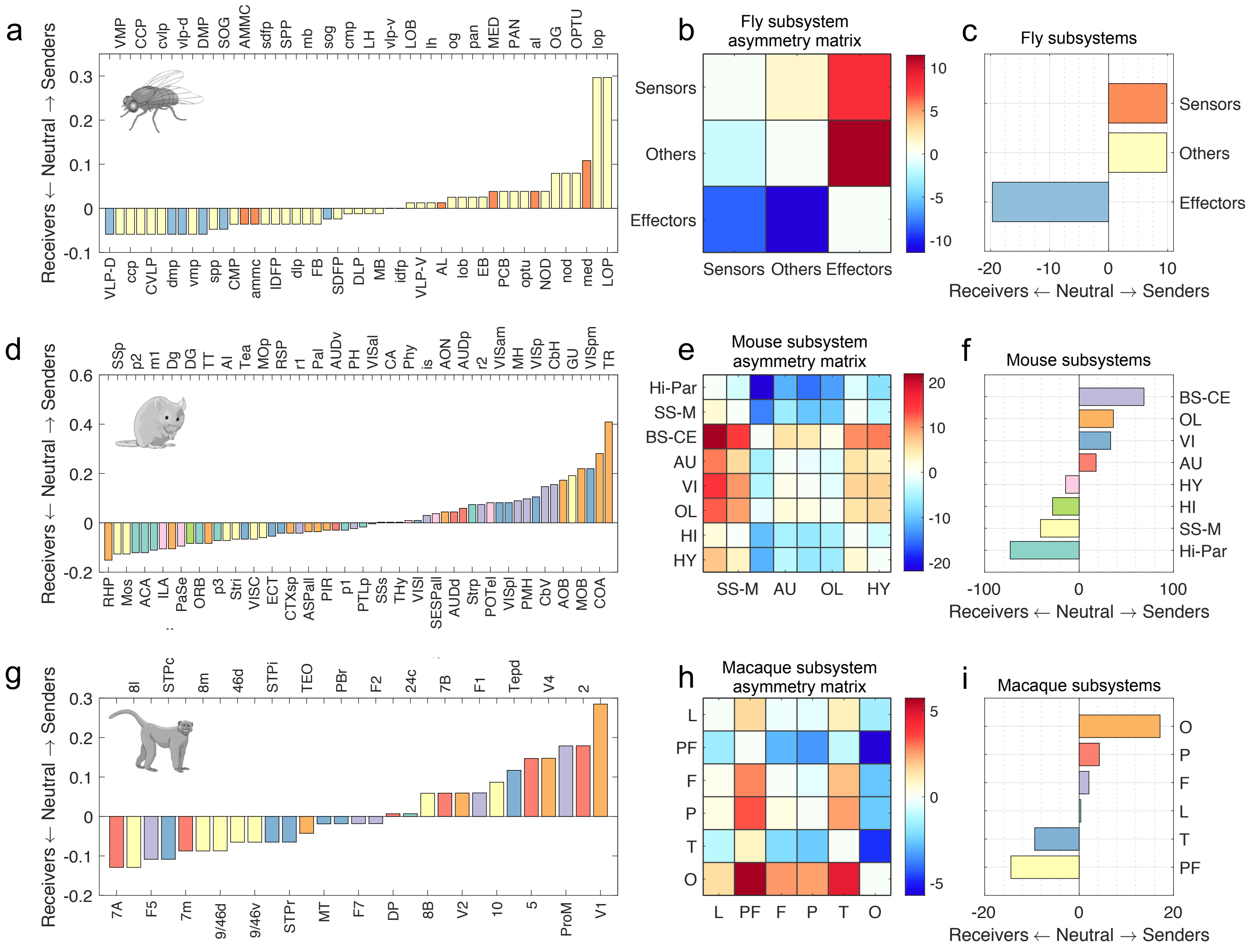

Supplementary Fig. 14. Send-receive asymmetry of undirected (symmetrized) non-human connectomes under binary diffusion efficiency. **(a)** Regions of the fly connectome sorted from receivers (negative send-receive asymmetry) to senders (positive send-receive asymmetry). Regions were divided into three subsystems [13], with sensor, effector and other nodes marked by orange, blue and beige bars, respectively. **(b)** Send-receive asymmetry matrix between subsystems of the fly connectome. For each subsystem pair i, j, we computed whether the mean of their distribution of node-level pairwise asymmetries was significantly larger than 0 by means of a one-sample t-test. Send-receive asymmetry between subsystems was defined as the resulting t-statistic. Red (positive t-statistic) and blue (negative t-statistic) indicate a bias towards communication efficiency in the $i \to j$ and $j \to i$ directions, respectively. **(c)** Fly subsystems sorted according to their send-receive asymmetry. Positive and negative values denote biases towards outgoing and incoming communication, respectively. **(d–f)** Same as (a–c) for the mouse connectome. Bar colors denote the affiliation of regions to previously identified subsystems [14]. **(g–i)** Same as (a–c) for the macaque connectome. Bar colors denote the affiliation of regions to previously identified subsystems [15].

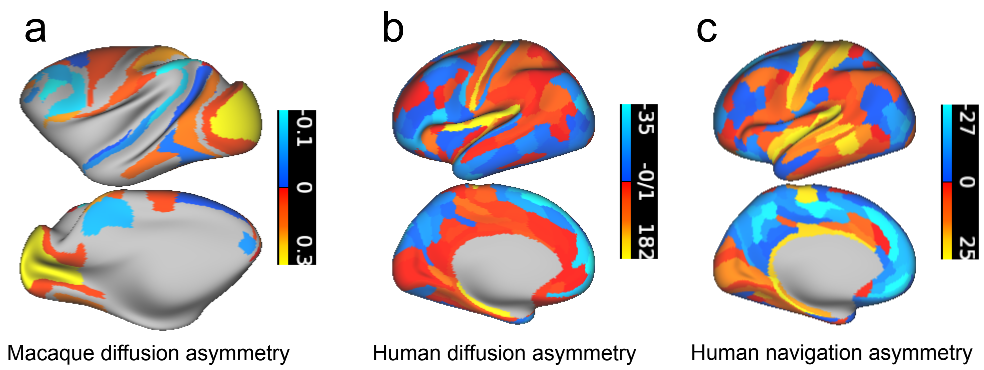

Supplementary Fig. 15. Comparison between the regional send-receive asymmetries for human and macaque. Orange and blue colors denote senders and receivers, respectively. **(a)** Binary diffusion asymmetry for the macaque. Gray regions are missing from the macaque connectome. **(b,c)** Weighted diffusion and navigation asymmetries for the human connectome.
