# Supplementary material for: Inferring neural signalling directionality from undirected structural connectomes
Source: Nat Commun. 2019 Sep 19;10:4289. doi: 10.1038/s41467-019-12201-w (PMC6753104; doi:10.1038/s41467-019-12201-w)
Supplement: Supplementary file 2 — Reporting Summary [file 41467_2019_12201_MOESM2_ESM.pdf]

nature research

Corresponding author(s): Caio Seguin

Last updated by author(s): Sep 11, 2019

# Reporting Summary

Nature Research wishes to improve the reproducibility of the work that we publish. This form provides structure for consistency and transparency in reporting. For further information on Nature Research policies, see Authors & Referees and the Editorial Policy Checklist.

## Statistics

For all statistical analyses, confirm that the following items are present in the figure legend, table legend, main text, or Methods section.

| n/a | Confirmed | |
|---|---|---|
| ☐ | ☒ | The exact sample size (*n*) for each experimental group/condition, given as a discrete number and unit of measurement |
| ☐ | ☒ | A statement on whether measurements were taken from distinct samples or whether the same sample was measured repeatedly |
| ☐ | ☒ | The statistical test(s) used AND whether they are one- or two-sided<br>*Only common tests should be described solely by name; describe more complex techniques in the Methods section.* |
| ☐ | ☒ | A description of all covariates tested |
| ☐ | ☒ | A description of any assumptions or corrections, such as tests of normality and adjustment for multiple comparisons |
| ☐ | ☒ | A full description of the statistical parameters including central tendency (e.g. means) or other basic estimates (e.g. regression coefficient) AND variation (e.g. standard deviation) or associated estimates of uncertainty (e.g. confidence intervals) |
| ☐ | ☒ | For null hypothesis testing, the test statistic (e.g. *F*, *t*, *r*) with confidence intervals, effect sizes, degrees of freedom and *P* value noted<br>*Give P values as exact values whenever suitable.* |
| ☒ | ☐ | For Bayesian analysis, information on the choice of priors and Markov chain Monte Carlo settings |
| ☒ | ☐ | For hierarchical and complex designs, identification of the appropriate level for tests and full reporting of outcomes |
| ☐ | ☒ | Estimates of effect sizes (e.g. Cohen's *d*, Pearson's *r*), indicating how they were calculated |

*Our web collection on statistics for biologists contains articles on many of the points above.*

## Software and code

Policy information about availability of computer code

| | |
|---|---|
| Data collection | All analyses in the present study were carried out on publicly available datasets. |
| Data analysis | Functions to compute navigation efficiency, diffusion efficiency and search information are available as part of the Brain Connectivity Toolbox (https: //sites.google.com/site/bctnet/). Further analy- ses and computations were performed using MRtrix3 (www.mrtrix.org/), SPM12 (https://www.fil.ion. ucl.ac.uk/spm/software/spm12/) or custom MATLAB code available at https://github.com/caioseguin/sen_rev. |

For manuscripts utilizing custom algorithms or software that are central to the research but not yet described in published literature, software must be made available to editors/reviewers. We strongly encourage code deposition in a community repository (e.g. GitHub). See the Nature Research guidelines for submitting code & software for further information.

## Data

Policy information about availability of data

All manuscripts must include a data availability statement. This statement should provide the following information, where applicable:

- Accession codes, unique identifiers, or web links for publicly available datasets
- A list of figures that have associated raw data
- A description of any restrictions on data availability

All analyses in the present study were carried out on publicly available datasets. Structural and effective human brain networks were mapped from Human Connectome Project data [30] (https://db.humanconnectome. org/). The fruit fly connectome was collated from data available in http://www.flycircuit.tw and can be found in the supplementary information of reference [44]. The macaque connectome was derived from data available at http://core-nets.org/ [47]. The mouse connectome was constructed from resources provided by the Allen Institute for Brain Science (https://mouse. brain-map.org/ [45]) and is available in the supplementary information of reference [46]. The cortical gradient of functional connectivity from reference [35] is available at https://www.neuroconnlab.org/data/index.html.
Functions to compute navigation efficiency, diffusion efficiency and search information are available as part of the Brain Connectivity

Toolbox (https: //sites.google.com/site/bctnet/). Further analy- ses and computations were performed using MRtrix3 (www.mrtrix.org/), SPM12 (https://www.fil.ion. ucl.ac.uk/spm/software/spm12/) or custom MATLAB code available at https://github.com/caioseguin/sen_rev.

# Field-specific reporting

Please select the one below that is the best fit for your research. If you are not sure, read the appropriate sections before making your selection.

☒ Life sciences ☐ Behavioural & social sciences ☐ Ecological, evolutionary & environmental sciences

For a reference copy of the document with all sections, see nature.com/documents/nr-reporting-summary-flat.pdf

# Life sciences study design

All studies must disclose on these points even when the disclosure is negative.

| | |
|---|---|
| Sample size | No sample size calculations were performed. |
| Data exclusions | No data were excluded from the analyses. |
| Replication | All attempts to replicate findings were successful. |
| Randomization | This is not relevant to this study since we do not consider multiple subject groups. |
| Blinding | Not relevant. See above. |

# Reporting for specific materials, systems and methods

We require information from authors about some types of materials, experimental systems and methods used in many studies. Here, indicate whether each material, system or method listed is relevant to your study. If you are not sure if a list item applies to your research, read the appropriate section before selecting a response.

## Materials & experimental systems

| n/a | Involved in the study | |
|---|---|---|
| ☒ | ☐ | Antibodies |
| ☒ | ☐ | Eukaryotic cell lines |
| ☒ | ☐ | Palaeontology |
| ☒ | ☐ | Animals and other organisms |
| ☒ | ☐ | Human research participants |
| ☒ | ☐ | Clinical data |

## Methods

| n/a | Involved in the study | |
|---|---|---|
| ☒ | ☐ | ChIP-seq |
| ☒ | ☐ | Flow cytometry |
| ☒ | ☐ | MRI-based neuroimaging |
